# Reactive gliosis in traumatic brain injury: a comprehensive review

**DOI:** 10.3389/fncel.2024.1335849

**Published:** 2024-02-28

**Authors:** Zuzana Amlerova, Martina Chmelova, Miroslava Anderova, Lydia Vargova

**Affiliations:** ^1^Department of Neuroscience, Second Faculty of Medicine, Charles University, Prague, Czechia; ^2^Department of Cellular Neurophysiology, Institute of Experimental Medicine of the Czech Academy of Sciences, Prague, Czechia

**Keywords:** traumatic brain injury, glia, experimental models, neuroinflammation, intercellular signaling, neurodegeneration

## Abstract

Traumatic brain injury (TBI) is one of the most common pathological conditions impacting the central nervous system (CNS). A neurological deficit associated with TBI results from a complex of pathogenetic mechanisms including glutamate excitotoxicity, inflammation, demyelination, programmed cell death, or the development of edema. The critical components contributing to CNS response, damage control, and regeneration after TBI are glial cells–in reaction to tissue damage, their activation, hypertrophy, and proliferation occur, followed by the formation of a glial scar. The glial scar creates a barrier in damaged tissue and helps protect the CNS in the acute phase post-injury. However, this process prevents complete tissue recovery in the late/chronic phase by producing permanent scarring, which significantly impacts brain function. Various glial cell types participate in the scar formation, but this process is mostly attributed to reactive astrocytes and microglia, which play important roles in several brain pathologies. Novel technologies including whole-genome transcriptomic and epigenomic analyses, and unbiased proteomics, show that both astrocytes and microglia represent groups of heterogenic cell subpopulations with different genomic and functional characteristics, that are responsible for their role in neurodegeneration, neuroprotection and regeneration. Depending on the representation of distinct glia subpopulations, the tissue damage as well as the regenerative processes or delayed neurodegeneration after TBI may thus differ in nearby or remote areas or in different brain structures. This review summarizes TBI as a complex process, where the resultant effect is severity-, region- and time-dependent and determined by the model of the CNS injury and the distance of the explored area from the lesion site. Here, we also discuss findings concerning intercellular signaling, long-term impacts of TBI and the possibilities of novel therapeutical approaches. We believe that a comprehensive study with an emphasis on glial cells, involved in tissue post-injury processes, may be helpful for further research of TBI and be the decisive factor when choosing a TBI model.

## 1 Introduction

Traumatic brain injury (TBI) is one of the most common types of brain injury, with major medical and socio-economic problems (Hyder et al., 2007; Rubiano et al., 2015). It is approximated that TBI annually afflicts a range of 54 to 60 million individuals, necessitating hospitalization or culminating in mortality. Worldwide, TBI accounted for 27.16 million new cases (with 95% uncertainty intervals of 23.36 to 31.42 million), 48.99 million prevalent cases (46.84 to 51.32 million), and 7.08 (5.00 to 9.59 million) million years lived with disability (YLDs) in 2019 (Guan et al., 2023). On a global scale, the age-standardized incidence rates of TBI have demonstrated a significant decrease since 1990. This positive trend suggests potential benefits arising from international initiatives aimed at lowering TBI rates. Notably, substantial efforts have been directed toward enhancing road safety and mitigating road-related injuries, a prominent contributor to TBI globally. Among the diverse injury categories, cerebral injuries exhibit a heightened propensity to resulting in either death or sustained disabilities (Hyder et al., 2007). The primary factors contributing to TBI vary by age, socio-economic conditions and geographical locations. Low and medium-income countries report an approximately threefold higher proportional incidence of TBI compared to high-income countries. The Southeast Asian and Western Pacific regions bear the highest overall burden in this regard (Dewan et al., 2018). According to studies, falls have been identified as the predominant etiological factor for TBI (74%), followed by road injuries (19%, mainly Africa and Southeast Asia), violence (2%, South America, Caribbean and Sub Saharan Africa), and sports- and work-related incidents (Iaccarino et al., 2018). Over 25% of individuals with long-term mild traumatic brain injury (mTBI) consequences struggle to return to work even a year post-injury (Langlois et al., 2006). Chronic mTBI represents a formidable health challenge characterized by lifelong disabilities and enduring consequences, significantly diminishing the affected individuals’ quality of life (Seabury et al., 2018). The economic ramifications are noteworthy; the estimated overall healthcare cost attributable to non-fatal TBI in 2016 was, with rehabilitation costs per patient reaching $36.000, contributing to an annual national expenditure of nearly $40.6 billion (Miller et al., 2021). Despite these profound implications, the molecular mechanisms underpinning chronic mTBI symptoms remain elusive within current scientific understanding.

Traumatic brain injury is a complex process, in which the primary injury followed by the secondary injury evokes pathological structural changes combined with functional deficits (Galgano et al., 2017). The term primary injury refers to an initial insult caused by mechanical forces, that depending on the intensity/severity and type of injury, may result in contusion or penetration of the brain and/or blood-brain barrier (BBB) disruption. The primary injury can evoke a focal or diffuse type of tissue damage. Diffuse injury occurs with a higher frequency than a focal one, however, a combination of both types is common in patients (Skandsen et al., 2010). The secondary tissue damage occurs over a period of time, from minutes up to several days after the primary injury, and is caused by cellular reactions, activation of metabolic pathways associated with neuroinflammation, edema, hypoxia, or tissue atrophy (Ng and Lee, 2019). The damage and the extent of the injury caused by TBI are determined by the severity of the injury, which can be assessed by various methods, including the Glasgow Coma Score (GCS). The GCS scale evaluates three parameters (eye, verbal, and motor responses) and based on the scores, the injury is classified as mild (GCS 13–15), moderate (GCS 9–12), or severe (GCS 3–8) (Jain and Iverson, 2022). The majority of reported TBI in patients is of mild level (Leo and McCrea, 2016), but numbers of cases may be higher as numerous patients with suspected mTBI do not seek medical help after an accident. Additionally, the period following TBI can be divided into three phases: acute (24 h post-TBI), subacute (1 day–3 weeks post-TBI), and chronic phase (more than 3 weeks post-TBI). However, these time periods are only general and may vary from case to case as they are affected by various factors, such as age, injury type and location (Lund et al., 2016; Toshkezi et al., 2018).

Secondary brain injury comprises a complex series of cascading events within the brain. These events include the release of pro-inflammatory cytokines, chemokines, and signaling molecules, which create a pro-inflammatory microenvironment. This environment exacerbates neuronal excitotoxicity and oxidative stress, contributing significantly to the development of secondary brain damage (Ng and Lee, 2019). Glial cells, representing the most abundant brain cells, play a prominent role in these events, as well as in synaptic pruning, synaptic plasticity alterations, and the long-term functional outcomes of the injured brain, emphasizing their central position in the pathophysiology of secondary brain injury (Mira et al., 2021).

In response to tissue trauma, glia cells undergo both morphological and functional changes characterized by the term reactive gliosis, which usually refers to astrocytes and microglia (Buffo et al., 2008; Gao et al., 2013; Pekny and Pekna, 2016). Reactive glia, as part of the lesion, are involved in neuroinflammation and edema to varying degrees, create the glial scar and produce extracellular matrix (ECM) molecules, such as chondroitin sulfate proteoglycans (CSPGs) that contribute to glial scar formation and stabilize it (Asher et al., 2001; Dyck and Karimi-Abdolrezaee, 2015). Moreover, reactive glia produce a large spectrum of proteins, including growth factors and cytokines that are crucial for intercellular cooperation as well as for protective mechanisms and regeneration of damaged tissue (Ziebell and Morganti-Kossmann, 2010).

In the healthy brain, **astrocytes** are the main effector cells responsible for the optimal environment for neuronal survival and functions: they maintain ion/pH/water homeostasis and BBB integrity, modulate synaptic activity, ensure the neurotransmitter clearance from the extracellular space and are involved in neuronal metabolism, by providing energy substrates as well as by metabolite removal (Kim et al., 2019). During TBI, besides being a key component forming glial scar, reactive astrocytes produce pro-inflammatory cytokines and chemokines (Gyoneva and Ransohoff, 2015; Michinaga and Koyama, 2021). Changes in the expression and localization of various astrocytic proteins forming ion channels and transporters, such as aquaporin-4 (AQP4) or glutamate transporters EAAT1/GLT-1 and EAAT2/GLAST, also contribute to edema development and excitotoxicity (van Landeghem et al., 2006; Beschorner et al., 2007; Kapoor et al., 2013). Unlike astrocytes, the functions of **microglia** are much more narrowly focused and their main function under physiological conditions is immune surveillance and the phagocytosis of apoptotic debris. However, it has also been demonstrated that microglia can be involved in synapse formation and maintaining synaptic homeostasis, as well as in the production of a variety of growth factors modulating neuronal activity (Borst et al., 2021). In the injured brain, microglia are the key components of neuroinflammation, and together with astrocytes they contribute to scar formation (Wake et al., 2009; Miyamoto et al., 2013). **Oligodendrocytes** are myelinating cells providing support and insulating myelin sheaths to axons. Pathologies such as TBI accompanied by oxidative stress or excitotoxicity have a detrimental effect on oligodendrocytes, resulting in their apoptosis and demyelination, which can significantly affect the resulting post-TBI functional outcome (Dent et al., 2015). There is increasing evidence of the important role of oligodendrocyte progenitor cells (OPCs), also known as polydendrocytes or **NG2-glia,** in brain pathologies, including TBI (Dimou and Gallo, 2015; Akay et al., 2021). NG2-glia have a large capacity to proliferate and differentiate into other cellular types, mostly in myelinating oligodendrocytes under physiological conditions (Zhu et al., 2008; Dimou and Gallo, 2015). In the injured brain, NG2-glia become part of the lesion and glial scar, contribute to wound closure, regulate neuroinflammation, and besides oligodendrocytes, NG2-glia can differentiate into other cell types, especially reactive astrocytes which seem to be injury type-dependent (Honsa et al., 2016; Hackett et al., 2018).

Traumatic brain injury research articles are mostly aimed at astrocytes and microglia, while NG2-glia and oligodendrocytes are usually neglected. In this review, we focus on the roles of these four types of glial cells in secondary injury pathologies based on observations in the different TBI models and factors that may impact the outcomes and should be considered when designing experiments.

## 2 Experimental models of traumatic brain injury

Traumatic brain injury is a highly variable and complex process, and there is currently no animal model which would be able to completely reproduce all the changes that occur after TBI. To date, a number of TBI models have been developed that differ in the type of the induced injury (focal vs. diffuse; impacted vs. non-impacted) as well as in the severity of the tissue damage. Here, we list a brief overview of the most known models: Weight drop injury (WD), Controlled cortical impact (CCI), Fluid percussion injury (FPI), Blast injury (BI), Penetrating ballistic-like brain injury (PBBI) and Close-head impact model of engineered rotational acceleration (CHIMERA) [for comprehensive reviews see (Xiong et al., 2013; McDonald et al., 2021)]. With regards to the necessary equipment, the WD model is the simplest to perform, while the others require sophisticated devices. Rodents are the most used animals for TBI experiments, but animals such as zebrafish (Hentig et al., 2021) and *Drosophila* (Katzenberger et al., 2013) are used as well. The CCI and WD models can be produced as an open-head or closed-head injury (CHI), FPI and PBBI are open-head type of models, while BI and CHIMERA are CHI models. Open head models require a craniotomy, and the impact is directed to the surface of the dura. CHI is a type of TBI, where the force impacts on the intact skull (Deshetty and Periyasamy, 2023).

### 2.1 Weight drop (WD)

**FIGURE 1:**
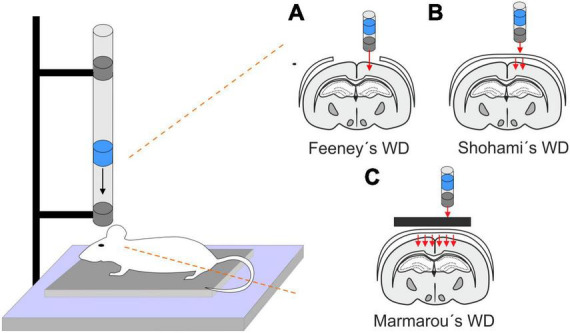
Models of traumatic brain injury - Weight drop (WD). WD models use free fall of guided weight in different modifications. **(A)** In Feeney’s WD model, the weight drops on the exposed dura. **(B)** In Shohami’s WD model, the weight impacts an unprotected scull. **(C)** In Marmarou’s WD model, the weight is dropped on a metal disc or other scull protection and simulates a diffuse concussive head injury for example in football players or car accidents.

This model is performed by a free fall of guided weight to an exposed skull (depending on the model, with or without craniotomy) (Figure 1). There are 3 basic types of this model: 1. Feeney’s model exposes the dura, and the weight impact results in cortical contusion followed by the development of a necrotic cavity (Feeney et al., 1981). 2. Shohami’s model is a CHI model, where weight impacts an unprotected skull. Due to this model, edema occurs in the traumatized hemisphere with the addition of hemorrhagic lesions which in turn develop into necrosis (Shapira et al., 1988). 3. The Marmarou’s model uses a metal disc to prevent skull fractions. This model mimics TBI induced by falls and motor vehicle accidents and evokes a diffuse injury without lesion development (Marmarou et al., 1994). The severity of the WD model can be adjusted by changes of the weight mass and/or height of the drop of the weight (Ma et al., 2019). The WD model replicates human TBI fairly well, and the WD device is simple and affordable in comparison to other models. However, complications with skull fractures, high mortality, and variability in observed injuries may emerge (Zhang et al., 2014).

### 2.2 Controlled cortical impact (CCI)

**FIGURE 2:**
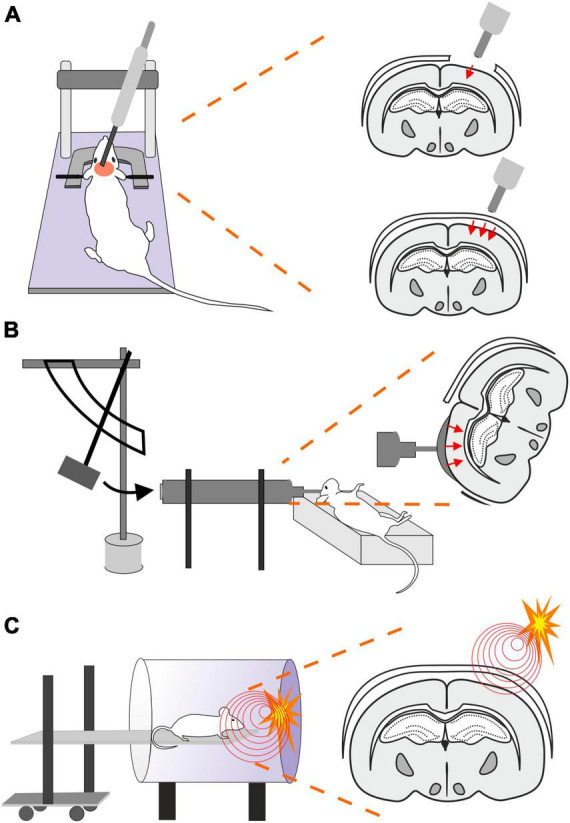
Models of traumatic brain injury–Controlled cortical impact (CCI), Fluid percussion injury (FPI) and Blast injury (BI). **(A)** In CCI model, pneumatic- or an electromagnetic-controlled piston impact the exposed dura. The severity of the evoked focal TBI depends on the depth of the impact and the velocity of the piston impacting either the exposed dura (frequently used as a model of focal/open head injury; upper scheme) or unprotected skull (modified CCI to mimick diffuse/closed head injury; lower scheme). Moreover, the TBI severity is also affected by the material of the pad (foam vs. solid) and impactor tip (rubber vs. solid) or by the presence or absence of head fixation. **(B)** Brain injury in FPI model is evoked by a fluid pulse transferred to exposed dura by the impact of a pendulum on a piston filled with fluid. This mode mimics brain contusion without scull fraction and its outcome depends on the localization of the craniotomy and the angle of the pendulum. **(C)** In the BI model, the animal is placed on a movable platform in a shock tube capable of controlling overpressure waves evoked by a controlled detonation. The model simulates diffuse mTBI often seen in military personnel.

This model uses a controlled piston (electromagnetic or pneumatic control) which delivers an impact to the exposed dura (Osier and Dixon, 2016; Figure 2A). This type of model evokes a focal brain injury resulting in cortical tissue loss, axonal injury, BBB disruption and development of edema. Moreover, this model was adapted to be used for close head injury, CCI-CHI (Romine et al., 2014; Osier and Dixon, 2016). The severity of the resulting injury can be adjusted by controlling the depth impact and the velocity of the piston (Ma et al., 2019).

The advantages of this model are the low mortality rate, no risk of rebound injury, and result reproducibility. However, it also depends on the impactor diameter and whether the simple CCI or adapted CCI-CHI is used (Zhang et al., 2014; Ma et al., 2019).

### 2.3 Fluid percussion injury (FPI)

The FPI model uses a fluid pulse delivered to the dura by the impact of a pendulum on a piston filled with fluid (Ma et al., 2019; Figure 2B). Based on the placement, this technique can be used for lateral FPI (a combination of focal and diffuse injury) or midline FPI (diffuse injury). FPI simulates brain contusion without skull fracture. Localization of the craniotomy is an important factor when using this model. Floyd et al. (2002) tested 4 groups with rostral, caudal, medial, and lateral craniotomy. The caudal group exhibited a higher mortality rate than other groups after FPI, and the rostral group showed the lowest level of cellular loss in the hippocampal area. The authors also showed differences in the levels of reactive astrogliosis in a brain area-dependent manner, whereas the rostral group showed less immunoreactivity for astrocytic marker glial fibrillary acidic protein (GFAP) than the rest of the groups. However, it should be noted that fewer animals per group were used for the GFAP expression tests than for the rest of the aforementioned examinations (Floyd et al., 2002). The FPI model fairly accurately mimics the glial reaction to TBI, as the studies using this model reported hypertrophy of astrocytes with different orientation, and a decreased number of their processes or structural changes of microglia (rod-like morphology) (Ziebell et al., 2012; Robinson et al., 2016). The FPI model can be adjusted by setting the angle of the pendulum higher or lower (Eakin et al., 2015). This model results in direct brain trauma without the necessity of skull protection, and the mortality rate is relatively low. The disadvantage of this model is the necessity of fluid percussion system monitoring during the procedure, since factors such as residual air in the system can cause variability or added injuries (Zhang et al., 2014).

### 2.4 Blast injury (BI)

Blast-induced traumatic brain injury (bTBI) is caused by exposure to explosive devices. This type of injury is often called “invisible injury” because there is no evidence of an external injury (Lucci, 2006). This form of neurotrauma represents the most prevalent type of mTBI in the context of military personnel. Blast waves cause significant oscillating acceleration-deceleration cycles on the head, which is known as the “bobblehead effect” (Rosenfeld et al., 2013). The detonation of an explosive device generates a supersonic pressure gradient, caused by a primary blast wave. This wave consists of a high positive pressure followed by a prolonged negative pressure phase, forming a Friedlander curve. Primary bTBI is characterized by internal injuries that are challenging to detect and assess for severity. Strong blasts can cause acute injury or death, while exposure to milder forces may result in delayed or subclinical neuropathological changes. To simulate bTBI, a shock tube capable of controlling overpressure waves is utilized to mimick real-life free-field explosions, resembling the Friedlander-type waveform (Huber et al., 2013; Campos-Pires et al., 2018; Figure 2C).

### 2.5 Penetrating ballistic-like brain injury (PBBI)

**FIGURE 3:**
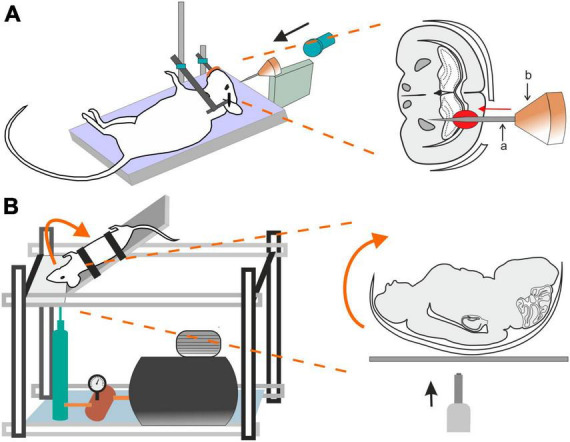
Models of traumatic brain injury–Penetrating ballistic-like brain injury (PBBI) and the Closed-head impact model of engineered rotational acceleration (CHIMERA). **(A)** In the PBBI model, a modified air-rifle is utilized to accelerate a projectile (led pellet) into a conic impactor probe (a) placed in an impactor holder (b) against the animal head which is fixed by stereotactic apparatus and a nose clip. Probe penetration results in the creation of temporary brain cavity, mimicking the damage after a gun shot. The severity of the injury can be modified by adjusting the air-rifle pressure. **(B)** In the device utilized for a mouse CHIMERA, a piston, driven by a high-pressure pulse, impacts the plate which the animal’s head is freely resting on, while the animal body is fixed in a supine position. The impact results in a sagittal plane motion of the animal head.

Penetrating ballistic-like brain injury (PBBI) results from high-energy projectiles, creating a substantial temporary cavity in the brain (Williams et al., 2005, 2006; Figure 3A). The outcome is closely linked to the projectile’s anatomical path and energy transfer (Williams et al., 2005). Using this model in rats results in cognitive impairment, white and gray matter damage, brain swelling, seizures, cortical spreading depression, and neuroinflammation (Williams et al., 2007). Studies using new rodent PBBI models, including a non-fatal model which uses a modified air rifle for low-velocity PBBI or another PBBI model simulating the effect of high-energy projectiles, reveal immediate and subacute changes such as increased intracranial pressure, BBB permeability, brain edema formation, and persistent motor and cognitive deficits (Plantman et al., 2012). The unique aspect of extensive intracerebral hemorrhage makes PBBI a valuable model for studying moderate-to-severe brain trauma as well as for assessing the effect of therapeutic interventions (Williams et al., 2006; Chen et al., 2009; Shear et al., 2009).

### 2.6 Closed-head impact model of engineered rotational acceleration (CHIMERA)

This model is highlighted for its relevance in studying the impact of the acceleration effects associated with major trauma, including falls, vehicular accidents, and sports-related collisions (Namjoshi et al., 2014) and is particularly important for modeling axonal and white matter injury (Bashir et al., 2020; Krieg et al., 2023). CHIMERA’s commercial availability is emphasized, providing researchers with the means to standardize injury parameters and ensure consistent replication across various laboratories. Specifically, the mouse-oriented CHIMERA device exhibits compressive contact and inertial forces, along with changes in velocity and angular velocity akin to values observed in professional football and boxing (Namjoshi et al., 2014).

The CHIMERA TBI model utilizes the high-pressure impact from a metal piston on the platform under the animal’s head (Figure 3B). While the animal head rests freely, its body is secured in a supine position with straps, aided by crosshairs for precise head alignment. Compressed air, regulated by a valve, propels the piston, and its velocity is measured by infrared devices. A pressure gauge regulates impact intensity. Upon device activation, the ascending platform initiates a forward swing of the animal head that touched the sternum before returning to the original position on the platform (McNamara et al., 2020). All CHIMERA models involve animals in a supine position, causing sagittal plane motion. CHIMERA device reconfiguration enables a comparison of sagittal and horizontal impacts, shedding light on varying functional and morphological outcomes associated with different injury planes (Browne et al., 2011; Mychasiuk et al., 2016).

### 2.7 Repeated mild traumatic brain injury

The protocols of WD, CCI, FPI, and BI can be adapted to produce only mTBI by adjustment of the velocity, depth of duration of a traumatic pulse/impact. To simulate the head trauma often seen in contact sports, models of repetitive mild traumatic brain injuries (rmTBIs) have been introduced, where each single injury is below the concussion threshold, but their effects add up and may result in prolonged and/or delayed tissue damage and neurological deficit. The parameters of experimental protocols and observed alteration in biochemical/neuropathological analysis as well as in neurological outcome in different studies is thoroughly described in a systematic review of Hiskens et al. (2019).

### 2.8 Pediatric traumatic brain injury models

Traumatic brain injury is a significant global issue in infants and children. Head injuries in children can lead to a variety of traumatic injuries to the scalp, skull, and brain, mirroring those in adults. The necessity to study developmental TBI and its long-term consequences in appropriate models arose from significant distinctions in blood flow, metabolic rate, neurotransmitter activity, degree of myelination, ability to tolerate oxidative stress, and/or biomechanical scull properties between adult and the pediatric population (Margulies and Thibault, 2000; Morrison et al., 2013; Araki et al., 2017). For simulation of pediatric TBI, already established modified models such as WD, FPI, CCI, CCI-CHI, CHIMERA, and rmTBI models are employed as well as specific models for abusive head trauma (AHT), commonly known as shaken baby syndrome, which is the predominant form of TBI in infants <1 year (Bonnier et al., 2004). Experimental animals include rodents aged 11–21 days, covering responses from a term infant (aged 7–11 days) to a toddler (aged 17–21 days), piglets (especially in FPI and CHIMERA models) and lambs (shake injury models). Further insights into this topic are provided by comprehensive reviews focusing on the considerable challenges in the field and highlighting essential models that investigate the unique injury mechanisms related to pediatric TBI (Araki et al., 2017; Kochanek et al., 2017; Nwafor et al., 2022).

## 3 The response of glial cells to traumatic brain injury

Both human brain trauma as well as its experimental models induce a typical response of nervous tissue that is based predominantly on glia reaction and include:

### 3.1 Edema

**FIGURE 4:**
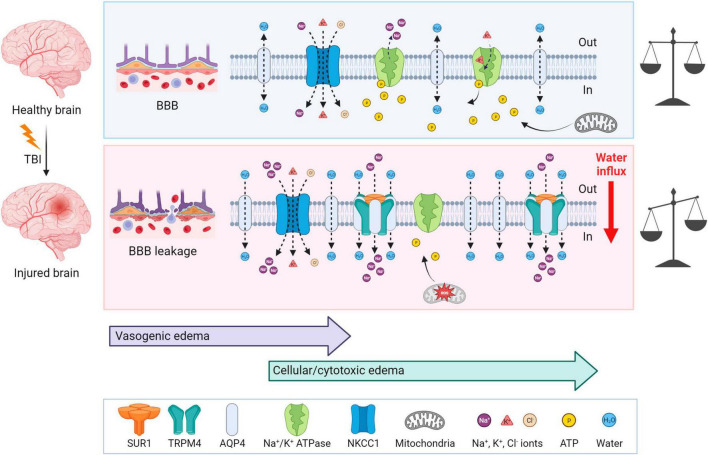
Mechanisms of brain edema development after traumatic insult—a comparison with a healthy brain. **Upper** panel: In physiological conditions, ionic balance is ensured by active or passive transport via channels, and mitochondria produce enough ATP to secure normal cellular function. **Lower** panel: At first, TBI induces BBB disruption and vasogenic edema, which is followed by cytotoxic edema with a slower onset. After TBI, AQP4, SUR1, and TRPM4 are upregulated and create a complex which contributes to astrocytic swelling by the intracellular transport of Na^+^ and H_2_O. NKCC1 during TBI is more activated and transports additional ions into the cells, particularly Na^+^. Moreover, due to the mitochondrial dysfunction induced by ROS, ATP is depleted. Lack of ATP inhibits the active transport via Na^+^/K^+^ ATPase and also terminates the ATP-dependent inhibition of SUR1-TRMP4 complex that transport Na^+^ ions. These events cause water influx and energetic and ionic disbalance, which can consequently result in cell death. TBI, traumatic brain injury; BBB, blood-brain barrier; NKCC1, Na^+^, K^+^, Cl^–^ cotransporter; ROS, reactive oxygen species; SUR1, sulfonylurea receptor 1; TRPM4, transient receptor potential melastin 4. Created with BioRender.com.

Traumatic brain injury is typically accompanied by cerebral edema, which is characterized by an increase and retention of CNS water content, contributing to elevated intracranial pressure. Two types of edema can be present post-TBI: vasogenic and cellular/cytotoxic edema. The vasogenic edema accompanies the compromised BBB integrity after TBI and it is characterized by an extracellular accumulation of fluid. The vasogenic type of edema prevails in the first days post-TBI; while the disrupted BBB gradually closes, cytotoxic edema with a slower onset follows (Donkin and Vink, 2010; Figure 4). Cytotoxic edema, the most common type of edema found in TBI patients (Marmarou et al., 2006), results in cellular swelling (including astrocytes), which can eventually result in apoptosis. However, this type of edema does not contribute to brain swelling or an increase in intracranial pressure.

Cytotoxic edema is characterized by cellular swelling, which arises from an intracellular influx of ions and water. The astrocytes express various channels contributing to water and ion homeostasis, such as aquaporins, NKCC1 (Na^+^, K^+^, Cl^–^ cotransporter), Na^+^/K^+^ ATPase, or SUR1-TRPM4 (Sulfonylurea receptor 1–transient receptor potential melastin 4) channels (Figure 4). ATP-dependent ion pump functionality is tightly connected with ATP availability. During TBI, generated reactive oxygen species (ROS) cause mitochondrial dysfunction leading to impairment in ATP synthesis and thus to ATP depletion. Moreover, ATP is released from cells into the extracellular space immediately following TBI, therefore exacerbating the energetic disbalance (Moro et al., 2021). The authors also demonstrated that this ATP release can be slowed down significantly by blocking P2Y1 receptors or store-operated calcium channels (Moro et al., 2021). Hence, the ATP-dependent Na^+^/K^+^ pump, creating membrane potential by maintaining the Na^+^/K^+^ difference across the membrane, fails in providing transport of the ions, causing a change in the membrane potential, followed by an increase in osmolarity and water influx into the cells (Zusman et al., 2020).

Aquaporin-4 (AQP4), a channel participating in water homeostasis and transport, is also considered to be an important contributor to cytotoxic edema. AQP4 is predominantly expressed by astrocytes and enriched in their endfeet in contact with blood vessels (Neely et al., 2001; Amiry-Moghaddam et al., 2004). TBI causes the upregulation of AQP4 expression, which consequently induces the swelling of astrocytes (Kapoor et al., 2013). The degree of edema is dependent on the subcellular localization of AQP4; translocation of AQP4 mediated by calmodulin leads to increased water flux and astrocytic swelling (Kitchen et al., 2020). In AQP4 knockout mice, the migration of astrocytes to the injury site was reduced (Saadoun et al., 2005). Additionally, in wild-type astrocytes, AQP4 is polarized to the leading edge of the membrane and astrocytic migration can be enhanced by the extracellular osmotic gradient. Therefore, authors suggested, that AQP4 is involved in astrocytic cell migration toward the damaged areas after TBI (Saadoun et al., 2005).

Other contributors to astrocytic swelling include the increased activation of NF-κB (Nuclear factor κB) (Jayakumar et al., 2014) and the increased activation of NKCC1 cotransporter in astrocytes, which controls the transport of Na^+^, K^+^ and Cl^–^ (Jayakumar et al., 2011). SUR1 is an ATP-binding part of various ion channels, serving as their regulatory subunit. SUR1 is associated with pore-forming subunits, such as Kir6.2 (ATP-sensitive potassium channel) or TRPM4 (ATP- and calcium-sensitive non-selective cation channel) (Chen et al., 2003). Interestingly, SUR1-TRPM4 is expressed in injured tissue, but not under physiological conditions (Chen and Simard, 2001; Chen et al., 2003). In human patients with post-traumatic brain contusions, overexpressed SUR1 was found in both astrocytes and microglia, and overexpressed Kir6.2 was observed in astrocytes (Martinez-Valverde et al., 2015; Castro et al., 2019). Additionally, upregulated SUR1, TRPM4, and Kir6.2 were discovered in both TBI patients and a CCI model of TBI (Gerzanich et al., 2019). ATP binding to SUR1-TRPM4 blocks its activity. Thus, ATP depletion leads to channel opening with consequential cation (predominantly Na^+^) influx, cell depolarization, and cellular swelling (Chen and Simard, 2001). Stokum et al. (2018) demonstrated an assembly of the SUR1-TRPM4-AQP4 complex in a murine model of brain edema using cerebellar cold injury (Stokum et al., 2018). Under physiological conditions, AQP4 creates a complex with other Na^+^ ion channels, such as TRPV4 (transient receptor potential cation channel subfamily V member 4) (Benfenati et al., 2011) or Na^+^/K^+^ ATPase (Illarionova et al., 2010). In the pMCAo (permanent middle cerebral artery occlusion) model of cerebral ischemia, loss of the AQP4-TRPV4 complex in double knockout mice leads to a reduction in the cytotoxic edema/ischemic lesion during the ischemic acute phase (Sucha et al., 2022). Overall, research in AQP4 and its complexes imply, that AQP4 and other channels, such as TRPV4 or TRPM4, may play a crucial role in cellular swelling and the extent of edema development in pathological states associated with AQP4/TRPV4/TRPM4 overexpression (Chmelova et al., 2019; Gerzanich et al., 2019; Sucha et al., 2022).

Following TBI, upregulated SUR1-TRPM4 and AQP4 assemble as a heteromultimeric water/ion channel complex, where SUR1-TRPM4 activity generates an osmotic pressure and causes water influx via AQP4 (Stokum et al., 2018; Figure 4). Interestingly, SUR1-Kir6.2 works contrarily to the SUR1-TRPM4 channel upon ATP depletion; activation of SUR1-Kir6.2 is followed by an outflux of K^+^ and hyperpolarization of the astrocytic membrane (Simard et al., 2012). To the best of our knowledge, there are no published studies explaining how TBI causes SUR1-Kir6.2 upregulation and which consequences it may cause, but some information is available from ischemia research. In the ischemic pMCAo model, SUR1-Kir6.2 was shown not to participate in cellular swelling while the SUR1-TRPM4 activity contributed to cytotoxic edema development (Woo et al., 2020). Additionally, some articles suggest the contribution of SUR1-Kir6.2 in a better outcome after hypoxia/ischemia (Yamada and Inagaki, 2005; Li et al., 2013).

Furthermore, the microglial immune response to TBI contributes to edema by the induction of AQP4 overexpression. Necrotic neurons from damaged tissue release HMGB1 (high-mobility group box protein 1) which in turn activates microglial TLR4 (Toll-like receptor 4). This event triggers a release of interleukin-6 (IL-6) from microglia, and as a result, it increases astrocytic AQP4 expression (Laird et al., 2014).

### 3.2 Excitotoxicity

**FIGURE 5:**
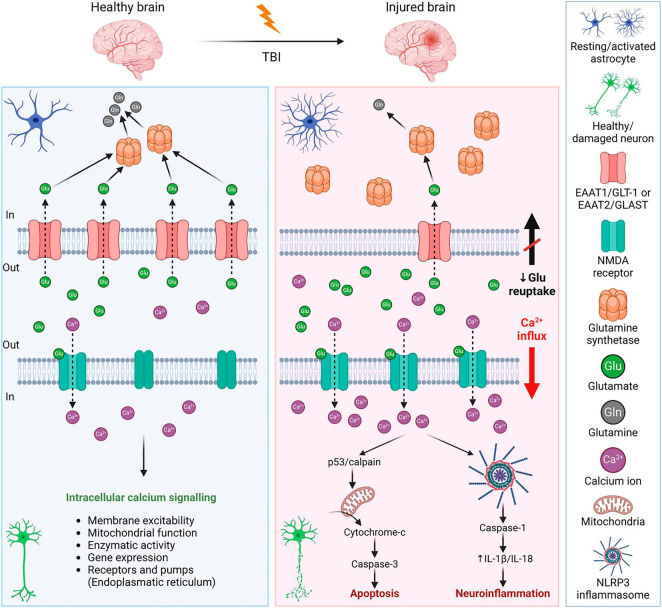
Mechanisms of glutamate excitotoxicity after traumatic brain injury—a comparison with a healthy brain. **Left** panel: In a healthy brain, the excess glutamate is cleared by astrocytes, where glutamate is converted into glutamine by glutamate synthetase. It is a strictly balanced environment to prevent neuronal death and neuroinflammation. **Right** panel: After TBI, the concentration of glutamate in the extracellular space increases due to disrupted BBB, or reduced glutamate reuptake due to the decreased expression of astrocytic glutamate transporters. Increased activation of neuronal NMDA receptors by glutamate binding evokes an influx of Ca^2+^ into the cells. Excessive Ca^2+^ triggers apoptotic signals leading to cell death and a release of IL-1β that induces immune response. For further details see text. NMDA receptor, *N*-methyl-D-aspartate receptor; IL-1β, interleukin 1β; BBB, blood-brain barrier; EAAT1/2, excitatory amino acid transporter 1/2; GLT-1, glutamate transporter 1; GLAST, glutamate aspartate transporter; NLRP3, NLR family pyrin domain containing 3. Created with BioRender.com.

Excitotoxicity is the process of exaggerated activation of the neuronal amino acid receptors (e.g., NMDA, AMPA, or KA receptors) by their excessive exposure to neurotransmitters, such as glutamate. Consequently, the influx of extracellular Ca^2+^ into neurons occurs, where Ca^2+^ may eventually trigger apoptotic signals through calpain mediating p53 induction and following caspase3-dependent neuronal apoptosis (Sedarous et al., 2003; Figure 5). Moreover, the Ca^2+^ influx induces neuroinflammation via NLRP3 (NLR family pyrin domain containing 3) and caspase-1. NLRP3 inflammasome is an intracellular sensor that detects microbial motifs and endogenous danger signals, providing a fast immune response (Kelley et al., 2019; Johnson et al., 2023). It is a complex composed of three protein subunit types: NLRP3 (a sensor protein), ASC (the adapter protein apoptosis-associated speck-like protein containing a CARD; caspase recruitment domain), and caspase-1. Besides altered calcium signaling, NLRP3 inflammasome is activated by other signals after TBI, including ionic changes (e.g., potassium and chloride efflux), or presence of extracellular ATP and ROS (O’Brien et al., 2020). After activation, NLRP3 inflammasome allows self-cleavage of pro-caspase-1 to active caspase-1, which consequently produces IL-1β or IL-18 (Johnson et al., 2023). The overexpression of IL-1β and IL-18 is followed by neuroinflammation that can lead to neuronal injury, apoptosis, or necrosis (Dong et al., 2009; Abdul-Muneer et al., 2017). Activation of NLRP3 inflammasome can also lead to cell death via pyroptosis (O’Brien et al., 2020; Figure 5).

After TBI, levels of extracellular glutamate increased in both patients and experimental models of TBI (Palmer et al., 1993; van Landeghem et al., 2001; Chamoun et al., 2010; Sowers et al., 2021). The excess glutamate contributes to secondary injury and increases the extent of the damage. There are different causes contributing to the elevated extracellular concentration of glutamate, which include increased release (disrupted BBB, dysregulated exocytosis) or decreased reuptake by glutamate transporters. Through glutamate transporters, both astrocytes and microglia participate in glutamate clearance. In the brain, there are several subtypes of glutamate transporters; glutamate transporters EAAT1/GLT-1 and EAAT2/GLAST are predominantly astrocytic, but they are present in microglia as well. Other glutamate transporters (EAAT3-5) are predominantly neuronal. In human patients with TBI, a decreased expression of EAAT1/GLT-1 and EAAT2/GLAST was observed (van Landeghem et al., 2006; Beschorner et al., 2007), which supports the idea of the involvement of glutamate transporters in the tissue damage and final outcome after TBI. Therefore, many studies have focused on glutamate metabolism and glial glutamate transporters.

Lehmann et al. (2009) used cultured cortical astrocytes to study the influence of high glutamate levels on the expression of glutamate transporters and glutamine synthetase (GS), which converts transported glutamate into glutamine. In their study, astrocytic expression of both EAAT1/GLT-1 and EAAT2/GLAST decreased in high glutamate medium (0.5–20 mM) via glutamate receptor-independent mechanism. On the contrary, an increase in GS expression was induced by higher levels of glutamate (≥1 mM) (Lehmann et al., 2009). A significant decrease in the protein expression of glutamate transporters was reported in the ipsilateral cortex during the acute phase following CCI in rats (Rao et al., 1998). In this model, ramified microglia with EAAT2/GLAST expression were present as early as 2 h post-TBI. From 4 up to 72 h after CCI, microglia expressing EAAT1/GLT-1 and EAAT2/GLAST were present in all observed areas, including the cortex, hippocampus, and lateral thalamus (van Landeghem et al., 2001). Yi et al. (2005) studied splice variants GLT-1v and GLT-1α in a rat model of lateral FPI. They observed a significant loss of GLT-1v (c-terminal splice variant) in the cerebral cortex (6–24 h post-TBI) and a transient decrease in the hippocampus and thalamus (6 h post-TBI). Interestingly, there was no significant change in GLT-1α in the cortex, but an increase was observed in the hippocampus (6–24 h post-TBI) (Yi et al., 2005). Additionally, it was found that GFAP (which is overexpressed after TBI) may modulate astrocytic glutamate transport in a region-dependent manner and be involved in EAAT2/GLAST trafficking (Hughes et al., 2004).

### 3.3 Neuroinflammation

Following TBI, the immune response is a natural process, which occurs within minutes post-injury. Although neuroinflammation is a necessary reaction to injury, its persistence causes long-term complications in TBI patients. Chronic neuroinflammation not only contributes to tissue damage but there is also growing evidence showing it as a main feature of other brain pathologies, such as dementia or Alzheimer’s disease (AD) (Eikelenboom et al., 2010; Perry et al., 2010). TBI itself is therefore considered a risk factor for AD and dementia development later in life (Plassman et al., 2000; Fleminger et al., 2003; Li et al., 2017), as persistent microgliosis is a common factor of these conditions.

**FIGURE 6:**
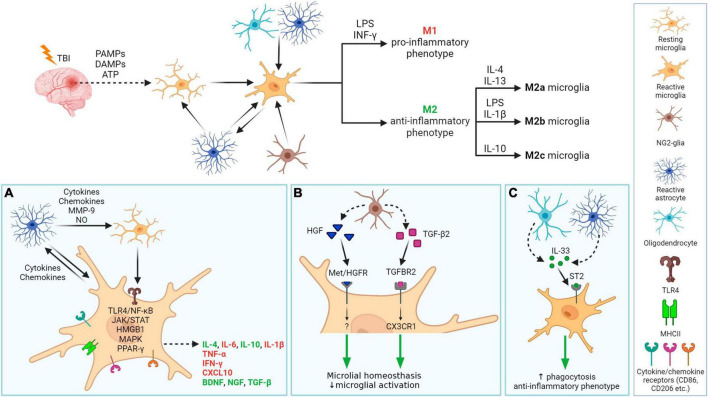
Neuroinflammation processes in damaged tissue after traumatic brain injury with a focus on glial cells. After TBI, PAMPs, DAMPs, and ATP are released from damaged tissue, which together with molecules (cytokines, chemokines) released by astrocytes, activate microglia. Activated microglia migrate into the damaged area and release pro- or anti-inflammatory factors. Microglial phenotype M1 and M2 (M2a-c resp.) phenotypes can be specifically activated. Other glial cells also have a role in neuroinflammation **(A–C)**. **(A)** In damaged tissue, cellular communication between activated astrocytes and resting or activated microglia occur. Activated microglia receive signals via their receptors that trigger one of the cascades, e.g., ligand binding to TLR4 induces NF-κB dependent pathway that evokes the release of IL-6, IL-1β, or TNF-α. . Released molecules can have pro-inflammatory (red) or anti-inflammatory properties (green). **(B)**: Microglial neuroinflammation activity can be regulated by NG2-glia, by releasing HGF of TGF-β2. **(C)** Both astrocyte and oligodendrocytes release IL-33, which promotes microglial anti-inflammatory phenotype and phagocytic ability. PAMPs, pathogen-associated molecular patterns; DAMPs, damage-associated molecular patterns; HGF, hepatocyte grow factor; TGF-β2, transforming grow factor β2; INF-γ, interferon γ; LPS, lipopolysaccharide; IL, Interleukin, e.g., IL-4; TLR4, toll-like receptor 4; MHCII, major histocompatibility complex class II; CD, cluster of differentiation, e.g., CD86; ST2, receptor suppression of tumorigenicity 2, also known as IL1RL1 or IL33R; interleukin-1 receptor-like 1, or interleukin-33 receptor; Met/HGFR, hepatocyte growth factor receptor encoded by the MET gene; TGFBR2, transforming growth factor beta receptor 2; CX3CR1, C-X3-C motif chemokine receptor 1; MMP-9, matrix metalloproteinase 9; NO, nitric oxide; TNF-α, tumor necrosis factor α; CXCL10, C-X-C motif chemokine ligand 10; BDNF, brain-derived neurotrophic factor; NGF, nerve growth factor; NF-κB, nuclear factor κB; JAK/STAT, janus kinase/signal transducers and activators of transcription; HMGB1, high-mobility group box 1; MAPK, mitogen-activated protein kinase; PPAR-γ, peroxisome proliferator-activated receptor γ. Created with BioRender.com.

Both microglia and astrocytes are involved in neuroinflammation, and their interplay is essential (Figure 6). Following TBI, microglia are activated by PAMPs (pathogen-associated molecular patterns) or DAMPs (damage-associated molecular patterns) released from damaged tissue (Jassam et al., 2017; Johnson et al., 2023). The process of microglial activation is complex and heterogeneous, depending on the severity, type of injury, and the extent of the damaged area and structure of the brain (Shields et al., 2020). In general, microglia express receptors recognizing cytokines, chemokines (e.g., CD86, CD206), and after activation, also major histocompatibility complex class II (MHCII). Microglia are activated by pro-inflammatory cytokines such as interferons (INF-γ), interleukins (e.g., IL-6), or tumor necrosis factor (TNF-α). Additionally, a fast microglial response is supported by ATP, released from damaged tissue, and by activated astrocytes (Davalos et al., 2005). Reactive astrocytes also contribute to microglial activation by producing cytokines, chemokines, nitric oxide (NO), or matrix metalloproteinase 9 (MMP-9). Furthermore, astrocytes, together with oligodendrocytes, secrete IL-33, which promotes the recruitment of microglia and macrophages post-TBI (Wicher et al., 2017). The production of inflammatory cytokines is mediated by TLR4 via TLR4/NF-κB pathway. During TBI, upregulation of TLR4 can be observed in hippocampal astrocytes and neurons and its depletion suppresses the production of pro-inflammatory cytokines IL-6, IL-1β, and TNF-α. Other pathways involved in signaling during neuroinflammation include JAK/STAT (Janus kinase/Signal transducers and activators of transcription), HMGB1, MAPK (Mitogen-activated protein kinase), or PPAR-γ (peroxisome proliferation-activated receptor γ). They are discussed in more details below.

Galectins act as master regulators in the inflammatory response associated with neurodegenerative disease development (Soares et al., 2021). Yip et al. (2017) found that activated microglia release galectin-3 as a TLR4 ligand, crucial for the full microglial response to proinflammatory stimuli, like lipopolysaccharide (LPS) (Yip et al., 2017). Furthermore, galectin-3 influences the inflammatory response to α-synuclein (α-syn) in microglial cells and act as a natural ligand of TREM2 (Triggering receptor expressed on myeloid cells 2) receptor involved in AD pathogenesis (Boza-Serrano et al., 2014; Qin et al., 2021). The role of galectin-3 has been intensively studied in ischemia and tumorogenesis, where it was shown to activate or modulate other signaling molecules and regulators of cellular processes such as interleukin kinase or tyrosine kinases (Wesley et al., 2013; Porebska et al., 2021). However, several studies indicate a significant early increase in galectin-3 expression also in various trauma models, including experimental TBI (Natale et al., 2003; Venkatesan et al., 2010). Yip et al. (2017) discovered significant galectin-3 expression in mouse cerebrospinal fluid (CSF) 24 h post-injury, likely stemming from a disrupted BBB (Yip et al., 2017). Galectin-3-dependent-TLR4 activation may contribute to prolonged microglia activation and sustained brain inflammation (Burguillos et al., 2015). However, another study revealed a positive correlation between human plasma galectin-3 levels and higher GCS scores (Shen et al., 2016). These findings suggest the potential role of galectin-3 as a biomarker in TBI, indicating a need for further exploration for clinical applications. In contrast, the other member of galectin family, galectin-1, suppresses activation markers, proinflammatory cytokines, and inducible nitric oxide synthase (iNOS) expression in microglia in inflamed CNS tissues, while its absence prompts classical microglial activation (Aalinkeel and Mahajan, 2016).

Following activation, microglia undergo polarization—they change their morphology into larger rounded ameboid cells, proliferate and migrate into the damaged region (Caplan et al., 2020). Activated microglia also start to release factors with pro-inflammatory/anti-inflammatory functions. The basic microglial phenotypes after activation can be distinguished into two types–the M1 type and the M2 type. M1 type is considered a pro-inflammatory phenotype, preferably producing pro-inflammatory compounds such as cytokines TNF-α, INF-γ, IL-1β, chemokines (e.g., C-X-C motif chemokine ligand 10, CXCL10), or ROS. Alternatively, the neuroprotective M2 phenotype produces anti-inflammatory molecules, such as interleukin IL-10 (which can also be expressed by the M1 phenotype, but to a lower extent) (Mills et al., 2000; Ziebell and Morganti-Kossmann, 2010). The M2 phenotype has 3 subtypes: M2a, M2b, and M2c, which differ in function, activation factor, and expression of specific markers (Zhang et al., 2022a). Microglial phenotype can be specifically activated: IFN-γ and LPS stimulate M1 phenotype; IL-4 and IL-13 trigger M2a phenotype; M2b phenotype is the result of LPS or IL-1β activation and IL-10 triggers M2c phenotype (Figure 6). Each of these phenotypes has a specific proteomic profile (Xu et al., 2017; Vergara et al., 2019). Notably, transcriptomic analysis of activated microglia revealed the limitations of these classifications—activated microglia rarely fit the classification perfectly; usually a mixed microglial population is observed. Furthermore, microglial gene expression is dynamic and time-dependent. Izzy et al. (2019) demonstrated the reduced ability of microglia to sense tissue damage in the early stage of TBI, which in a later stage (14 days post-injury, dpi) began to transfer into a specialized inflammatory state, with changes of IL-4, IL-10, and IFN-γ gene expression within 14–60 dpi (Izzy et al., 2019). Due to this diversity, Paolicelli et al. (2022) oppose dual nomenclatures, such as M1/M2 or pro- vs. anti-inflammatory microglia. Instead of restricting to these types, the authors advise using descriptions of phenotypes and transcriptome (Paolicelli et al., 2022).

Until recently, it was thought that only astrocytes and microglia contribute to neuroinflammation. However, recent studies have revealed the important role of NG2-glia in the brain immune system as well as its involvement in neuroinflammation (Figure 6). Nakano et al. (2017) reported that the elimination of NG2-glia activates the IL-1β pro-inflammatory pathway resulting in defects of hippocampal neurons due to neuroinflammation. Furthermore, NG2-glia-expressed hepatocyte growth factor (HGF) acts as a regulator of the neuroinflammation level (Nakano et al., 2017). According to Zhang et al. (2019), transforming growth factor beta receptor 2 (TGF-β2) expressed by NG2-glia regulates the microglial chemokine receptor CX3CR1 (C-X3-C motif chemokine receptor 1) via increased phosphorylation of the transcriptional modulator mothers against decapentaplegic homolog 2 (SMAD2) and therefore controls microglial homeostasis. In any case, the deficiency of NG2-glia in the Parkinson’s disease (PD) mouse model contributed to neuroinflammation (Zhang et al., 2019). These studies suggest that NG2-glia may have a role in neuroinflammation regulation in both physiological and pathological states.

### 3.4 Cell death, demyelination, and white matter degradation

Cellular death typically accompanies TBI and was observed in both TBI patients and experimental models. Cellular death was documented in several brain regions, including the cortex, hippocampus, and thalamus (Clark et al., 1997; Conti et al., 1998; Fox et al., 1998). Apoptosis following TBI is triggered by a caspase-dependent pathway (Yakovlev et al., 1997; Clark et al., 2000) or a caspase-independent pathway, where apoptosis is induced by several mitochondrial proteins, e.g., mitochondrial apoptosis-inducing factor AIF (Zhang et al., 2002). Apoptosis is region dependent, with an early response in white matter (12 h post-TBI) and a delayed apoptosis peak in the thalamus (2 weeks post-TBI) (Conti et al., 1998). Neurons and oligodendrocytes in white matter are more vulnerable to apoptosis following TBI than the rest of the cells in the brain. Loss of oligodendrocytes is supplied by NG2-glia proliferation, which can be observed even months after injury (Dent et al., 2015), and the differentiation into oligodendrocytes can be driven by myelin damage (Hill et al., 2014). Johnson et al. (2013a) found ongoing white matter degradation in the corpus callosum, and persistent neuroinflammation in patients 3 months post-injury (Johnson et al., 2013a). Flygt et al. (2016) observed oligodendrocyte cell death and an increase in the number of oligodendrocyte progenitor cells/NG2-glia in patients with moderate to severe TBI (Flygt et al., 2016). Oligodendrocyte cellular death is caused by several factors—the microglial release of inflammatory cytokines, such as TNF-α or IFN-γ, higher oxidative stress, increased levels of extracellular ATP and/or excess of glutamate, which commonly occurs in TBI tissue. Interestingly, Lotocki et al. (2011) demonstrated a positive effect of hypothermia on TBI-induced oligodendrocyte loss—a decrease in temperature after injury for 4 h led to suppression of caspase-3 activity, which consequently enhanced oligodendrocyte survival (Lotocki et al., 2011).

Since oligodendrocytes produce myelin, a decrease in the amount of myelin after TBI could be the result of oligodendrocyte apoptosis. Both white matter loss and demyelination can be detected in various brain regions. Damaged areas with atrophy include the corpus callosum (Johnson et al., 2013a; Green et al., 2014; Briggs et al., 2016), hippocampus (Green et al., 2014) pons or cerebellum (Spanos et al., 2007). TBI activates IKK/NF-κB (IKK, inhibitory-κB kinase activates NF-κB by phosphorylating IκBα, the inhibitory subunit of NF-κB) signaling in oligodendrocytes, which induces their senescence and is followed by disturbed myelination (Schlett et al., 2023). Mahoney et al. (2022) used magnetic resonance imaging to demonstrate that activated intracortical demyelination has a distinct spatial profile in comparison to the acute phase. Additionally, the impact of TBI-related demyelination manifests worse outcomes than age-related demyelination, where temporal, cingulated, and insular regions showed a higher degree of myelin loss (Mahoney et al., 2022). Demyelination is spontaneously followed by remyelination; this process has a protective effect ensuring axonal survival (Irvine and Blakemore, 2008). However, the remyelination process is limited by inflammation on the site of the demyelinated lesions and inadequate precursor differentiation.

Microglia, together with macrophages, remove dying cells, but the alleviation of inflammatory responses may also enhance apoptotic processes. According to Wang et al. (2020), microglial depletion promoted neurite growth and reduced the total neural apoptosis after the FPI model of injury (Wang et al., 2020). The microglial pro-inflammatory response can directly trigger apoptosis of oligodendrocytes; conversely the anti-inflammatory M2 phenotype of microglia support remyelination (Miron et al., 2013), therefore a balance between M1 and M2 phenotype seems to be essential for the remyelination process. Blocking of microglial NHE1 (Sodium-hydrogen antiporter 1), the protein responsible for NADPH oxidase and cytokine secretion in pro-inflammatory microglia, resulted in a reduced inflammatory response, and consequently promoted oligodendrogenesis and supported remyelination in TBI tissue (Song et al., 2022).

### 3.5 Reactive gliosis and scar formation

The formation of a scar following TBI is considered a defense mechanism, preventing the propagation of the tissue damage and the spread of toxic metabolites; however, it also prevents axonal growth leading to impaired neural function recovery (Silver and Miller, 2004; Burda and Sofroniew, 2014; Karve et al., 2016).

**FIGURE 7:**
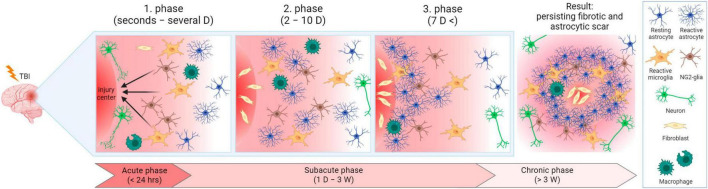
Lesion development after traumatic brain injury in time. In the acute phase, cells are damaged in the injury center, and immune cells are activated and recruited. Microglia and NG2-glia migrate toward the injured area and astrocytes become reactive. In the subacute phase, cells (including scar-forming astrocytes) proliferate. A layer of reactive astrocytes surrounds a forming lesion core. During the transition between the subacute and chronic phases, tissue remodeling and lesion maturation occur, resulting in permanent fibrotic and astrocytic scars. D, day/days; W, week/weeks. Created with BioRender.com.

After TBI, it is plausible to divide the ensuing events into three phases, while the processes of these phases can overlap (Burda and Sofroniew, 2014; Figure 7). In the first phase (seconds to hours after injury, prolonged up to several days), trauma evokes acute cell death in the injury center. Immune cells are activated and recruited, including microglia. Microglia and NG2-glia migrate toward the damaged area, astrocytes do not migrate, but swell and can eventually become reactive. During the second phase (2–10 days after injury), cells, including scar-forming astrocytes, proliferate. During this phase, a lesion core containing fibroblasts, pericytes, or inflammatory cells, starts to form. The core is surrounded by a layer of reactive astrocytes separating the core and the peri-lesion perimeter, which contains reactive glial cells, and gradually transitions to healthy tissue. Tissue remodeling appears in the third phase (which can occur 7 dpi) and the lesion matures. The result is a persisting fibrotic and astrocytic scar. Tissue remodeling is also present in the peri-lesion perimeter and can persist for months post-TBI (Burda and Sofroniew, 2014).

However, a mild diffused injury may result in gliosis without scar core formation (Shandra et al., 2019). In various articles, the term “glial scar” or “astroglial scar” is used for the whole lesion. Therefore, it was suggested not to use these terms because astrocytes are not mesenchymal or stromal cells, which form scars in other damaged tissues (e.g., skin or heart). Hence, “scar formation” occurring in the brain after TBI does not correspond to the widely used terminology from other research areas (Sofroniew, 2020). In contrast, other studies use the term “glial scar” only to describe the glial border of the scar since glial cells are not the main compounds of the lesion (Adams and Gallo, 2018).

NG2-glia together with microglia are the first glial cells to react to the injury, though microglia extend their processes faster than NG2-glia (Hughes et al., 2013). During injury, NG2-glia re-enter the cell cycle, which in combination with the shortening of the G1 phase, results in their proliferation and an increase of the NG2-glia population (Simon et al., 2011). OPCs, which belong to the NG2-glia population, are recruited via TLR-2/CXCR3 pathway signaling to the lesion site, where they differentiate into oligodendrocytes (Sanchez-Gonzalez et al., 2022). However, part of the NG2-glia form the glial border together with astrocytes and microglia, without any differentiation (Levine, 1994). Since the reduction of the NG2-glia population delays wound closure, it was suggested that accumulation of NG2-glia on the lesion site also has a positive effect on tissue regeneration (von Streitberg et al., 2021).

By releasing signaling molecules, including cytokines (TNF-α, IL-1β, IL-6) or IGF-1 (Insulin-like growth factor-1, in spinal cord injury), microglia stimulate the transformation of astrocytes into reactive astrocytes, which are a key element of the glial scar. Increased microglial proliferation reduces the lesion size and enhances functional recovery after spinal cord injury. On the contrary, microglial depletion evokes the suppression of astroglial STAT3 phosphorylation, a critical regulator of astrogliosis, resulting in impaired glial scar formation (Bellver-Landete et al., 2019; Zhou et al., 2023).

In astrocytes, TBI enhances proliferation, alters gene and protein expression and triggers cytoskeletal remodeling, resulting in hypertrophic astrocyte soma and processes. There are two essential proteins for astrocytic remodeling, GFAP and vimentin (Vim), which are both overexpressed post-TBI. GFAP and Vim are responsible for the assembly and extension of the intermediate filament inside the astrocytic processes, and astrocytes seem to suppress neurogenesis in the damaged tissue by producing these two proteins (Wilhelmsson et al., 2004). According to Jiang et al. (2018) expression of GFAP can be mediated by TLR4—the TLR4 deficit leads to suppression of GFAP upregulation and consequently repressed astrocytic activation (Jiang et al., 2018). Another important astrocytic protein, debrin, which is also overexpressed in the injured brain, controls scar formation via the regulation of membrane trafficking of crucial membrane receptors, such as β1-integrin (Schiweck et al., 2021).

Glial fibrillary acidic protein is frequently used as a marker of reactive astrocytes for immunofluorescence analysis. However, Escartin et al. (2021) do not consider GFAP as a reliable marker for evaluating the level of reactive astrogliosis, because different studies have shown that the level of GFAP in astrocytes varies (Shandra et al., 2019; Early et al., 2020; Escartin et al., 2021). Some studies even identified “GFAP-negative” astrocytic populations (Walz and Lang, 1998; Kofler et al., 2002; Xu, 2018). However, the GFAP expression level of “GFAP-negative astrocytes” may simply be under the detection limit, which is then observable due to injury-induced GFAP upregulation or caused by the masking of GFAP during the paraformaldehyde fixation of samples, specifically in gray matter (Walz, 2000). Nevertheless, GFAP is still a useful marker of reactive astrogliosis, although it is recommended to confirm the findings by the use of another marker.

Based on the type of upregulated proteins produced by activated astrocytes, Liddelow et al. (2017) suggested distinguishing two basic types of reactive astrocytes: A1 and A2, similarly, to the proposed microglia M1 and M2 phenotypes (Liddelow et al., 2017). The A1 phenotype includes neurotoxic astrocytes with upregulated pro-inflammatory factors, such as INF-γ, TGF-β, or IL-1α, or the activation of the NF-κB pathway, which have detrimental effects on the injured CNS. On the other hand, the A2 phenotype astrocytes display neuroprotective and anti-inflammatory effects, with upregulation of trophic factors BDNF (Brain-derived neurotrophic factor), VEGF (Vascular endothelial growth factor), or bFGF (Basic fibroblast growth factor) (Liddelow and Barres, 2017; Liddelow et al., 2017). Nevertheless, compounds produced by activated astrocytes may play both detrimental and protective roles. For example, IL-6 is a proinflammatory cytokine, that is overexpressed during TBI and has a key role in mediating neuroinflammation (Yang et al., 2013; Edwards et al., 2020; Ooi et al., 2022). In contrast, astrocytic overexpression of IL-6 is beneficial for wound closure and causes a decrease in oxidative stress and apoptosis in the TBI cryo-lesion model (Penkowa et al., 2003). However, upregulated IL-6 may also increase BBB permeabilization (Rochfort and Cummins, 2015) or promote cerebral edema (Xu et al., 2014). In recent years, studies using two-photon imaging and RNA-seq profiling have revealed much broader heterogeneity of the astrocytic population that does not fit with a simple A1/A2 type division (Adams and Gallo, 2018; Early et al., 2020). Therefore, Escartin et al. (2021) recommend describing the astrocytic phenotype in combination with transcriptome analysis to avoid strict classification (Escartin et al., 2021). Despite the above-mentioned objections, some of the new articles still use the A1/A2 division.

Newly proliferated and elongated astrocytes were found to be a part of the glial scar border (Wanner et al., 2013). Reactive astrocytes increase the production of CSPGs, which also become part of the lesion. The degree of astrogliosis is dependent on the distance from the insult area (Wanner et al., 2013), with the highest level of astrogliosis observed in the injury site (Castejon, 1998). Due to the wide astrocytic heterogeneity, it is still not perfectly clear how exactly each subpopulation responds to the TBI.

Since the formation of the lesion has long-term consequences for patient health, the cells and molecules contributing to the formation of the lesion are potential targets for the treatment of TBI. It is generally believed that one of the factors preventing full recovery of the damaged tissue is the barrier created by astrocytes that prevents axonal regrowth. Alternatively, Anderson et al. (2016) showed that by preventing astroglial scar formation, no axonal growth was restored in the injured spinal cord. Interestingly, levels of CSPGs, known inhibitors of axonal growth (Asher et al., 2001, 2002), were not significantly altered, indicating that other cells are replacing the astrocytic production of ECM post-TBI (Anderson et al., 2016). However, astrocytes are not the only glial cell type that limits recovery—the accumulation of NG2 cells in the lesion together with the NG2 overexpression also inhibits axonal growth *in vitro* (Levine, 1994; Chen et al., 2002; Tan et al., 2005). On the contrary, Yang et al. (2006) showed, that NG2-glia promoted axonal growth *in vitro* and provided an adhesive substrate for axonal growth cones both *in vitro* and in the developing corpus callosum. The authors also demonstrated that upregulation of NG2 expression does not inhibit axonal growth *in vitro* (Yang et al., 2006). These findings emphasize the need for further research, to better comprehend the specific mechanisms through which glial cells impact post-injury recovery, and to resolve the contradictory observations in this field.

## 4 Important signaling pathways and factors regulating tissue response after traumatic brain injury

**FIGURE 8:**
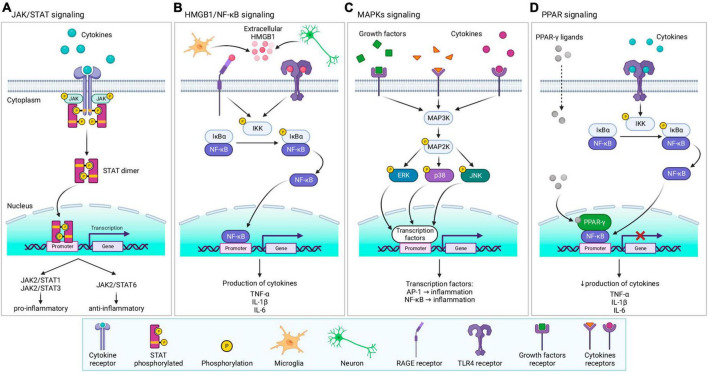
Overview of signaling pathways and factors involved in tissue response following TBI. **(A)** JAK/STAT signaling—after ligand binding to the receptor, JAK, and its downstream target STAT are activated. Activation triggers STAT dimerization, and translocation to the nucleus, where STAT dimer regulates the transcription of related genes, leading to the production of pro- or anti-inflammatory protein expression. **(B)** HMGB1/NF-κB signaling–extracellular HMGB1 released from microglia and neurons activates receptors (RAGE or TLR4), triggering the NF-κB pathway. IKK kinase phosphorylate IκBα, acts as an inhibitor of NF-κB which causes degradation of IκBα by the proteasome and translocation of NF-κB to the nucleus. After binding to the promoter, NF-κB regulates the transcription of related genes, leading to the production of cytokines. **(C)** MAPKs signaling—various ligands (cytokines, growth factors, etc.) can bind to specific receptors, triggering MAPKs cascade. Stimulation of MAP3K evokes activation of MAP2K, followed by activation of MAPKs (ERK, p38, or JNK) by phosphorylation. Activated MAPKs translocate into the nucleus and activate nuclear factors, including AP-1 or NF-κB, thereby regulating the transcription of related genes. **(D)** PPAR-γ signaling–specific ligands activate PPAR-γ, which consequently inhibits NF-κB, downregulating the transcription and decreasing inflammatory cytokines production. For further information see text. JAK, janus kinase;STAT, signal transducers and activators of transcription; HMGB1, high-mobility group box protein 1; NF-κB, nuclear factor κB; RAGE, receptor for advanced glycation end products; TLR4, toll-like receptor 4; IKK, inhibitory-κB kinase, also known as IκB kinase, IκBα, NF-κB inhibitor α; MAPK, mitogen-activated protein kinase; MAP3K, MAPK kinase; MAP2K, MAPK kinase; ERK, extracellular signal-regulated kinase; JNK, c-Jun N-terminal kinase; AP-1, activator protein 1; PPAR-γ, peroxisome proliferator-activated receptor γ; TNF-α, tumor necrosis factor α; IL, interleukin. Created with BioRender.com.

Neuroinflammation, and later neurodegenerative processes, are regulated by a number of signaling molecules and pathways whose detailed description would be beyond the scope of this review. Selected pathways and factors that are particularly involved in TBI-induced tissue response are discussed in more detail here (Figure 8):

### 4.1 Janus kinase/signal transducers and activators of transcription (JAK/STAT)

Janus kinase is a family of cytoplasmic non-receptor tyrosine kinases, which are involved in signal transduction with various downstream targets (Figure 8A). Mammalian JAK comprises four members (JAK1-3 and tyrosine kinase 2, Tyk2). One of their many downstream targets are STATs, which are a family of intracellular transcription factors, including seven members (STAT1-4, STAT5a/b, and STAT6). Upon ligand binding (e.g., cytokines) to a specific receptor, JAK is recruited in close proximity to the receptor and is activated by phosphorylation. Consequently, downstream substrate STAT is also activated by phosphorylation, causing STAT dimerization and its translocation from the cytosol to the nucleus, where STAT regulates the expression of the related genes (Rane and Reddy, 2000; Xin et al., 2020; Jain et al., 2021; Li et al., 2022b). The JAK/STAT pathway is involved in cell proliferation, differentiation, apoptosis, and mediating innate and adaptive immunity (Rane and Reddy, 2000; Xin et al., 2020; Jain et al., 2021; Li et al., 2022a). Signaling via the JAK/STAT pathway promotes either the pro-inflammatory M1 phenotype (JAK2/STAT1 and JAK2/STAT3) or the anti-inflammatory M2 phenotype (JAK2/STAT6) (Li et al., 2022a). Cornel iridoid glycoside (CIG), which inhibits the JAK/STAT phosphorylation (JAK1/3, STAT1/3) in microglial murine cell line BV2, also inhibits microglial polarization toward M1 phenotype, promoting polarization to M2 phenotype (Qu et al., 2019).

In the pericontusional cortex of the rat WD model, levels of phosphorylated JAK2, STAT1, and STAT3 increased, with the highest detected level 3 h post-injury (Zhao et al., 2011). Wang C. et al. (2023) showed that Myricetin, a flavonoid able to attenuate levels of pro-inflammatory cytokines *in vitro* (Martinez-Coria et al., 2023), decreased levels of phosphorylated STAT1 and STAT3, and increased levels of EGFR (Epidermal growth factor receptor) and phosphorylated AKT (Protein kinase B) in Myricetin-treated microglia after LPS induction (Wang C. et al., 2023). The authors also found lower levels of IL-1β, IL-6, and TNF-α, and increased IL-4 and IL-10 levels in CCI animal models treated with Myricetin, in comparison to injured animals without treatment. Overall, Myricetin suppresses the pro-inflammatory response after TBI via the EGFR-AKT/STAT pathway (Martinez-Coria et al., 2023; Wang C. et al., 2023).

### 4.2 High mobility group box 1 (HMGB1)

High mobility group box 1 is a non-histone DNA-binding protein that has localization-dependent functions. Nuclear HMGB1 plays a role in gene expression, DNA repair, and chromosome stability, and cytoplasmatic HMGB1 induces autophagy. However, extracellular HMGB1 acts as a pro-inflammatory cytokine/trigger (Paudel et al., 2020; Dong et al., 2022). Following injury, including TBI, production of HMGB1 is upregulated, and can be released into the extracellular space by both neurons and glia (active release or due to cell death), which binds to receptors, such as RAGE (Receptor for advanced glycation end products) or TLR4 (Figure 8B). Receptor activation leads to the production of cytokines via the NF-κB pathway (Paudel et al., 2018, 2020; Manivannan et al., 2021; Dong et al., 2022).

In recent years, the emerging role of HMGB1 in TBI has revealed the potential of HMGB1 as both a biomarker and target for TBI treatment (Paudel et al., 2018; Manivannan et al., 2021; Li et al., 2022b). In a CCI mouse model, Webster et al. (2019) showed that levels of extracellular HMGB1 are age-dependent, with higher extracellular as well as serum levels of HMGB1 in juvenile animals (aged 3 weeks) than in adults (aged 8-9 weeks) (Webster et al., 2019). To our knowledge, there is no article exploring HMGB1 expression and localization in the aged population after TBI.

Additionally, HMGB1 upregulation induced by the NLRP3 inflammasome contributes to cognitive dysfunction in the chronic phase of TBI (Tan et al., 2021). Inhibition of HMGB1 translocation caused a reduction of edema and production of TNF-α and iNOS in an FPI model (Okuma et al., 2012). Targeting HMGB1 also led to lower expression of IL-1β, TNF-α, and IL-6, and contributed to edema reduction in a CCI model of TBI (Yang et al., 2018). Inhibition of HMGB1 or RAGE causes a decrease in the number of pro-inflammatory microglia and a shift toward anti-inflammatory phenotype in spinal cord injury (Fan et al., 2020).

### 4.3 Mitogen-activated protein kinases (MAPKs)

Mitogen-activated protein kinases are a family of serine/threonine protein kinases, that convert extracellular stimuli into various cellular responses. Members of MAPKs involved in neuroinflammation and expression of inflammatory cytokines, include MAPK subfamilies ERK (extracellular signal-regulated kinase); ERK (1-7), p38 (p38α, p38β, p38γ and p38δ), or JNK (c-Jun N-terminal kinase; JNK1-3) (Cuenda and Rousseau, 2007; Figure 8C). MAPKs are activated by phosphorylation upon diverse signals, including the presence of inflammatory cytokines and glutamate toxicity (p38 and JNK) or the presence of growth factors, oxidative stress, and an intracellular influx of calcium (ERK) (Roux and Blenis, 2004; Cargnello and Roux, 2011; Otani et al., 2011).

Following TBI, albumin can be released and activate astrocytes and microglia via MAPK signaling. In study of Ralay Ranaivo and Wainwright (2010), albumin-activated astrocytes showed elevated levels of phosphorylated ERK, p38, and JNK, which decreased after 24 h to levels comparable with controls. Albumin also evoked upregulated production of IL-1β, nitrite, iNOS, and CX3CL1, and downregulated production of S100B (S100 calcium binding protein B) in astrocytes. Inhibition of p38 MAPK or ERK also decreases astrocytic production of IL-1β, nitrite, and CX3CL1, but does not change the levels of S100B. Microglia exposed to albumin contained increased levels of phosphorylated ERK, p38, and JNK as well as more upregulated production of IL-1β and nitrite than controls. Inhibition of microglial p38 MAPK (but not ERK or JNK) enhanced the IL-1β levels even more (Ralay Ranaivo and Wainwright, 2010). According to Mori et al. (2002), trauma activated ERK and p38 MAPK, but not JNK in both *in vitro* and the CCI model. The authors also showed that only the inhibition of ERK signaling increased cell survival *in vitro* and led to a decrease in lesion volume *in vivo*; inhibition of p38 or JNK did not show any effects (Mori et al., 2002). However, in the lateral FPI model, increases in the levels of phosphorylated ERK and JNK were detected, but not p38 (Otani et al., 2002). These discrepancies may be caused by differences in methodology or detection, or due to differences between TBI models.

The main p38 MAPK isoform responsible for regulation of cytokine production, p38α, was shown to upregulate the expression of IL-1β and TNF-α in LPS-induced BV2 microglial cell line (Cuenda and Rousseau, 2007; Bachstetter et al., 2011). Similarly, deletion of microglial p38α led to diminished neuroinflammation, alterations in microglial morphology, and downregulation of pro-inflammatory factor expression (IL-1β, IL-6, TNF-α, CCL2, CXCL10) in p38α knockout mice after CCI (Morganti et al., 2019). Interestingly, knockout of p38α evoked a significant increase in cytokine levels 6 h post-injury, in comparison to the wild-type midline FPI model. However, at 7 dpi cytokine levels were elevated only in the injured wild-type (Bachstetter et al., 2013). This study suggested, that p38α may be responsible for balancing the inflammatory response—reducing microglial overreaction in the acute phase and sustaining microglial activation in the later TBI phases.

Since MAPKs have an important role in neuroinflammation, its members are already known targets for anti-inflammatory drugs. Li et al. (2022a) studied the effects of curcumin on suppressing inflammatory processes in a WD model. Curcumin administration caused a decrease of phosphorylated p38 and NF-κB, and consequently a decrease in expression of pro-inflammatory cytokines IL-1β, IL-6, and TNF-α compared to untreated animals after injury (Li et al., 2022a). Kodali et al. (2023), administered human mesenchymal stem cell-derived extracellular vesicles (hMSC-EV) intranasally to animals after CCI. The hMSC-EVs were incorporated into neurons and microglia; the treatment inhibited the activation of NLRP3 inflammasome in the acute phase and reduced the density of pro-inflammatory microglia in the chronic phase. The authors also focused on molecules involved in p38/MAPK signaling—the treated animals showed a significant decrease in levels of IL-6, MyD88 (Myeloid differentiation primary response 88), AP-1 (Activator protein-1), and IL-8 compared to the injured animals, but levels of p38 MAPK did not differ between treated and injured animals (Kodali et al., 2023). Another drug used for the regulation of postmenopausal osteoporosis, Bazedoxifene, showed neuroprotective properties via inhibition of the MAPK/NF-κB pathway in the CCI model. Bazedoxifene suppresses phosphorylation of ERK, p38, and JNK, attenuates brain edema, and decreases levels of IL-6, IL-1β, COX-2 (Cyclooxygenase 2), and TNF-α (Lan et al., 2019).

### 4.4 Peroxisome proliferation-activated receptors (PPARs)

Peroxisome proliferation-activated receptors are a family of nuclear receptors, which have three members (PPAR-α, PPAR-β/δ, and PPAR-γ). In inflammatory conditions, PPARs role is to suppress the activity of NF-κB, which regulates the activity of immune cells, reduces the production of anti-inflammatory compounds (e.g., TNF-α or IL-1β), or diminishes oxidative stress (Cai et al., 2018; Figure 8D). In the context of TBI, PPAR-γ is the most extensively studied; due to its important and relatively well-known role, PPAR-γ is a focal point in neuroinflammation therapy. With the exception of inflammation, PPAR-γ also plays a role in the regulation of adipogenesis, glucose homeostasis, cellular differentiation, and apoptosis (Stahel et al., 2008; Qi et al., 2010; Cai et al., 2018; Korbecki et al., 2019).

Deng et al. (2020) found decreased levels of PPAR-γ, and increased levels of pro-inflammatory/apoptotic factors (IL-6, caspase-3, NO) in human TBI patients, and a similar decrease in PPAR-γ levels in an animal CCI model (Deng et al., 2020). The same study showed that application of Pioglitazone (a drug used to treat diabetes) in injured animals increased levels of PPAR-γ in comparison to both injured non-treated and control animals. Pioglitazone treatment also decreased levels of IL-6, and phosphorylated NF-κB, reduced cerebral edema, and improved functional outcomes (Deng et al., 2020). Additionally, application with a PPAR-γ antagonist worsened the neuroprotective properties of Candesartan (Angiotensin II AT1-receptor blocker) in the CCI model; the PPAR-γ antagonist caused an increase in the number of activated microglia and partially diminished the positive effect of Candesartan in lesion volume reduction (Villapol et al., 2012). These data suggest that Candesartan also activates PPAR-γ. Wu et al. (2023) tested the effect of ω-3 polyunsaturated fatty acid administration on the WD model of TBI. The authors found reduced levels of NF-κB, IL-1β, IL-6, and TNF-α in the treated animals, as well as reduced edema. Additionally, both mRNA and protein levels of PPAR-γ increased and NF-κB decreased in treated animals post-TBI, in comparison to injured animals without treatment. These results suggest that the neuroprotective outcome of ω-3 polyunsaturated fatty acid therapy is partially caused by affecting the PPAR-γ/NF-κB pathway (Wu et al., 2023).

### 4.5 Glia maturation factor (GMF)

Glia maturation factor is a protein expressed in various organs and cell types. GMF has two isoforms: a CNS-specific isoform GMF-β (GMFB), expressed by neuronal and glial cells, and GMF-γ (GMFG), expressed predominantly by endothelial and inflammatory cells. Despite its name, GMFG does not act as a glial maturation factor (Walker, 2003; Ikeda et al., 2006; Fan et al., 2018).

GMFB is a pro-inflammatory protein that contributes to neuroinflammation following injury, including TBI. GMFB promotes reactive gliosis after TBI, and regulates the polarization of activated microglia, with favor toward pro-inflammatory phenotype (Yin et al., 2018; Ahmed et al., 2020).

In the model of cryogenic TBI, GMFB protein levels increased by 1 dpi, with a maximum level 14 dpi in the lesion area. The injury also elevated mRNA levels of GMFB 7 dpi, with a maximum at 14 dpi (Hotta et al., 2005). Ahmed et al. (2020) found that the absence of GMFB results in a reduction of lesion volume, an increase in neuronal survival, and inhibited gliosis after WD, compared to wild-type injured animals. In contrast with the wild-type WD model, activated microglia polarized to anti-inflammatory phenotype in GMFB knockout animals post-injury, with a decreased production of TNF-α and IL-6 and an increased expression of IL-4 and IL-10 (Ahmed et al., 2020). Similarly, GMFB deletion reduced neuroinflammation, decreased phosphorylation of NF-κB, and downregulated the production of GFAP, iNOS, and COX-2 compared to injured wild-type animals (Selvakumar et al., 2020).

Since GMFB is associated with neuroinflammation, researchers also explored the potential role of GMFB as a modulator of inflammatory signaling pathways. *In vitro* studies showed that overexpression of GMFB can activate p38, and NF-κB, slightly induce ERK, and have no effect on JNK activation (Zaheer and Lim, 1998; Zaheer et al., 2001, 2007). GMFB phosphorylated by PKA (proteinkinase A) activates p38 but inhibits the activation of ERK *in vitro* (Lim and Zaheer, 1996; Zaheer and Lim, 1996). However, the exploration of connections between GMFB and inflammatory signaling pathways requires more in-depth investigation, incorporating *in vivo* experiments into the research.

## 5 Reactive species and free radicals in TBI-induced tissue damage and neurodegeneration

Oxidative metabolism represents a notable metabolic advancement for aerobic organisms as it produces 36 ATP molecules from each glucose molecule in the presence of oxygen (Reiter et al., 2017). The mitochondrial electron transport chain (ETC) generates ATP but also leads to electron leakage, resulting in the production of superoxide anion radicals (•O_2_^–^). Under physiological conditions, 1–3% of oxygen is converted to •O_2_^–^. However, disruptions in ETC components during CNS pathologies increase electron leakage and elevate ROS generation (Hiebert et al., 2015; Reiter et al., 2017).

Reactive oxygen species comprise mostly of free oxygen radicals containing at least one atom of oxygen together with one or more unpaired electrons. Besides •O_2_^–^ itself, ROS also include secondary reactive species generated by the reaction of •O_2_^–^ with other molecules (Cornelius et al., 2013; Fridovich, 2013), for example hydrogen peroxide, hydroxyl, peroxyl and hydroperoxyl radicals. ROS are vital for some processes involving energy transfer, immune protection and signaling (Hakiminia et al., 2022). During physiological conditions, antioxidant defenses, involving enzymes superoxide dismutase (SOD), glutathione peroxidase (GPx), catalase (CAT), glutathione reductase (GR), and non-enzymatic elements (glutathione, vitamin E, vitamin C), serve as effective safeguards against oxidative threats (Fernandez-Gajardo et al., 2014; Mendes Arent et al., 2014). Oxidative stress occurs when oxygen and free radicals surpass the antioxidant system’s capacity (Lewen et al., 2000).

Following injury-induced excitotoxicity, excessive Ca^2+^ levels promote the generation of ROS and NO (Higgins et al., 2010). Elevated free radical concentrations result in the modification of key macromolecules such as DNA, proteins, and lipids, impairing various cellular processes (Valko et al., 2007). The reversible and irreversible changes in these macromolecules contribute to a spectrum of disorders, including neurodegenerative disease (Droge, 2002; Poprac et al., 2017). Following TBI induction, the injured nervous system experiences dynamic changes with the emergence of potential •O_2_^–^ sources in the initial minutes and hours. These sources include enzymatic processes in the arachidonic acid (AA) cascade, autoxidation of biogenic amine neurotransmitters, “mitochondrial leak,” xanthine oxidase activity, and extravasated hemoglobin oxidation. Furthermore, NADPH oxidases (Nox), a family of membrane enzymes, actively convert oxygen into ROS. Nox enzymes significantly contribute to the pathophysiology of the nervous system, playing a pivotal role in the development of secondary injury following TBI (Angeloni et al., 2015). In the initial phase of TBI, an excess of ROS is predominantly generated by granulocytes and macrophages, rather than by activated or resting microglia, resulting in pronounced oxidative stress at the lesion site (Abe et al., 2018; Wu et al., 2022). During the chronic stage of TBI, microglia or macrophages assume a more prominent role in ROS production and oxidative stress within the injured brain, as evidenced by the augmented presence of Nox2 in microglia or macrophages 1 year post-injury (Loane et al., 2014).

Another significant category of reactive molecules constitute reactive nitrogen species (RNS), where Arginine (Arg) serves as their primary source. Arg is a semi-essential amino acid playing a crucial role as a precursor for metabolites such as ornithine, agmatine, creatine, and polyamines (Madan et al., 2018). Moreover, Arg metabolism, facilitated by nitric oxide synthases (NOS), generates NO and L-Citrulline. These metabolites contribute to diverse physiological functions, such as modulating cerebral blood flow, energy production, and tissue regeneration (Mader and Czorlich, 2022).

Depleting the L-Arg substrate can induce oxidative stress through the disconnection of NOS, resulting in the formation of oxygen radicals and potentially giving rise to the highly toxic oxidant peroxynitrite (ONOO^–^) (Cherian et al., 2004). NO possesses anti-inflammatory properties through redox regulation of the nuclear factor NF-κB (Reynaert et al., 2004). Consequently, the depletion of NO may intensify neuroinflammatory reactions. A decrease in Arg levels has been previously documented in the rat cortex 6 h after TBI using a WD model (Amorini et al., 2017). Furthermore, several clinical and preclinical studies have reported comparable changes in Arg metabolism lasting up to 3 dpi (Jeter et al., 2012; Zheng et al., 2020).

In experimental models, administering L-Arg after injury has demonstrated therapeutic benefits, including increased cerebral blood flow and reduced contusion volume. This positive effect is attributed to elevated NO production (Madan et al., 2018). However, literature on TBI presents conflicting roles of neuronal and glial NO. Inhaled NO increased cerebral blood flow and prevented ischemia causing secondary brain damage after TBI in mice, thus improving outcomes with prolonged exposure (Terpolilli et al., 2013). However, higher NO metabolites near neurons correlated with poorer survival in TBI patients (Tisdall et al., 2013). A placebo-controlled human trial with the NOS inhibitor VAS203 yielded better Glasgow Outcome Scale (categorizes the outcomes of patients after TBI) results at 6 months (Terpolilli et al., 2009). The inhibitor N(G)-nitro-L-arginine methyl ester (L-NAME), targeting all NOS forms, yielded mixed outcomes in TBI (Lu et al., 1997; Wada et al., 1999). These contradictions may result from spatial and temporal variations in NO production (Cherian et al., 2004). NO, generated from Arg by NOS isoforms, exhibits cell-specific expression: eNOS (endothelial) in vascular endothelium, nNOS (neuronal) in neurons, and iNOS (inducible) in macrophages and glial cells (Madan et al., 2018). Pharmacological investigations into NO’s role in TBI pathology have often used systemic approaches with L-Arg, a substrate for all NOS forms, or non-selective NOS inhibitors. However, such methods lack insights into the specific impact of L-Arg on distinct cell types or NO production from major cell-specific NOS isoforms (Madan et al., 2018).

Transcription factor Nrf2 (Nuclear factor erythroid-derived 2 related factor 2), acts as a regulator of both cytosolic and mitochondrial ROS production through NADPH oxidase, and is an important player in the oxidative stress-mediating tissue response following TBI (Kovac et al., 2015). Nrf2 signaling decreases with age in both humans and experimental animals, which suggests the possibility of an impaired response to oxidative stress in aged brains (Ngo and Duennwald, 2022).

Following activation by TBI-induced oxidative stress, Nrf2 is translocated from the cytoplasm into the nucleus, where it forms a dimer with transcription factor sMAF (small MAF protein; musculoaponeurotic fibrosarcoma) and then binds to ARE (Antioxidant response element, a *cis-*acting enhancer element), activating transcription of downstream targets, including heme oxygenase-1 (HO-1), glutathione S-transferase (GST), or SOD, which serve as protective enzymes against oxidative stress (Wu et al., 2022). Specifically, HO-1 catalyzes heme degradation that generates iron ions, biliverdin, and carbon monoxide (CO). In addition to the antioxidative role of biliverdin and CO, these products can also act as anti-inflammatory, anti-apoptotic, or anti-proliferative (Loboda et al., 2016). Morita et al. (2020) found an increased number of HO-1 expressing astrocytes (a significant increase in the number of HO-1-positive (HO-1^+^) cells at 1 dpi, with the maximum at 7 dpi) and microglia (delayed onset compared to astrocytes, maximum of HO-1^+^ cells at 14 dpi) in the CCI model (Morita et al., 2020). Furthermore, the NF-κB activity influences the ROS levels, by inducing the expression of both antioxidant (e.g., CAT, or HO-1) and pro-oxidant proteins (e.g., iNOS, or COX-2). ROS itself can influence NF-κB signaling at multiple levels of the pathway (both as an activator and inhibitor of the pathway) (Morgan and Liu, 2011).

Additionally, the cross-talk between Nrf2 and NF-κB is important for maintaining cellular homeostasis, because Nrf2 inhibits the NF-κB pathway and vice versa. Nrf2 and NF-κB compete for the co-activators of transcription, including the CREB binding protein (binding is dependent on the relative amount of Nrf2 and NF-κB in the nucleus), which can impact the balance and change the cellular inflammatory and stress response. The NF-κB p65 subunit can also directly inhibit the Nrf2 pathway. Interestingly, Rac-1 (Rac family small GTPase-1) activated NF-κB can induce Nrf2 and upregulate the expression of HO-1. Products of HO-1 then act as negative regulators of NF-κB inflammatory activity, by preventing NF-κB activation (Bellezza et al., 2018; Gao et al., 2021).

## 6 The role of exosomes and microRNA in reactive gliosis

The initiation and development of reactive gliosis is a complex process requiring precise communication between different cell types in which information is carried by the content of released extracellular vesicles (EVs). The family of EVs encompasses a diverse array of particles, including exosomes (30–100 nm diameter), microparticles (MPs; 100–1000 nm) and apoptotic bodies (2–5 μm) (Kumar et al., 2017). EVs have a dual role in cellular processes—they can be received by cells and simultaneously, they can be released from cells to transmit biological messages to neighboring cells. Exosomes are secreted by various cell types in the central as well as peripheral nervous system, including astrocytes (ADEs–astrocytes-derived exosomes), microglia (MDEs), oligodendrocytes (ODEs) and neurons (NDEs) (Potolicchio et al., 2005; Kramer-Albers et al., 2007; Thompson et al., 2016). The exosomes encapsulate diverse bioactive molecules such as microRNA (miRNA), mRNA and proteins (cytokines) and take part in intercellular crosstalk in both, physiological and pathological conditions (Kalluri and LeBleu, 2020).

In 2009, several expression profiling studies highlighted the potential role of miRNA in TBI (Lei et al., 2009; Redell et al., 2009). Notably, 136 miRNAs exhibited altered expression post-TBI, with changes evident across all four examined time points, except for miRNA-21. Bioinformatic analysis of validated miRNAs (miR-107, -130a, -223, -292-5p, -433-3p, -451, -541, and -711) regulated by TBI revealed an enrichment of proteins involved in diverse biological processes and functions typically initiated post-injury. These encompassed signal transduction, transcriptional regulation, proliferation, and differentiation (Redell et al., 2009). The list of miRNA with a known effect in TBI-induced gliosis can be found in Supplementary Table 2.

Interestingly, miRNA-21 expression and processing are governed by bone morphogenetic protein (BMP) and JAK/STAT signaling pathways, both of which have demonstrated a crucial role in regulating astrocytic responses (Setoguchi et al., 2004; Sahni et al., 2010; Kohanbash and Okada, 2012).

Sahni et al. (2010) revealed that the overexpression of miRNA-21 in cultured astrocytes induces a decrease in cell size, process thickness, and GFAP expression. These observations suggest a plausible role for miRNA-21 in modulating astrogliosis (Sahni et al., 2010). The upregulation of miRNA-21 expression is observed in astrocytes proximate to the lesion site following injury. Astrocytic hypertrophy decreases with miRNA-21 overexpression but increases with miRNA-21 inhibition, leading to more axon sprouting in the lesion during chronic injury stages (Bhalala et al., 2012). Targeting miRNAs may affect astrocytic hypertrophy, modulate glial scar progression and potentially improve functional recovery after brain injury.

## 7 The remote tissue response to traumatic brain injury

Glial responses to brain injury can be observed in a variety of brain structures and regions (Zhao et al., 2019). The following studies described TBI-evoked gliosis in the entorhinal cortex (Bolton and Saatman, 2014), hippocampus (Allen et al., 2000; Aungst et al., 2014; Bolton and Saatman, 2014; Luo et al., 2014; Zhao et al., 2017), corpus callosum (Bennett et al., 2012; Luo et al., 2014; Rodriguez-Grande et al., 2018; Zhao et al., 2019) and contralateral cortex (Miyazawa et al., 1995; Zhao et al., 2019).

In young mice, a single moderate TBI can result in long-lasting changes in astrocytes (GFAP and AQP4 staining) and microglia (Iba1 staining; Ionized calcium-binding adaptor molecule 1) in remote areas. Even 12 months after the damage, astrogliosis was still evident in the hippocampus; and both basal nuclei and the hippocampus continued to exhibit active microglia. Additionally, changes in the expression level and cellular location of the astrocytic protein AQP4 are dependent on the area. According to Obenaus et al. (2023), these findings have also been linked to cognitive deficiencies (Obenaus et al., 2023). The loss of AQP4 polarity brought on by TBI was demonstrated by Zhao et al. (2017) using the CCI mouse model. Even 4 weeks after the TBI, they observed astrogliosis in the contralateral hippocampal CA1 region. The GFAP-positive (GFAP^+^) reactive astrocytes soma and coarse processes are where AQP4 relocated, and this movement is controlled by adenosine A_2*A*_ receptor signaling. As a result of decreased phosphorylated tau clearance, AQP4 seems to shift in polarity, which is followed with hyperphosphorylated tau protein accumulation in the CA1 hippocampus (Zhao et al., 2017).

The result of an injury may vary if the damage occurs in gray or white matter only or in both. Mattugini et al. (2018) identified and defined macrophage migration inhibitory factor (MIF) as a factor that regulates NG2-glia proliferation. The upregulation of MIF in injured gray matter supports the proliferation of NG2-glia, but not astrocytes and/or microglia. On the other hand, down-regulation of MIF is induced, if both gray and white matter are injured. The authors suggest that the proliferation of glial cells in gray matter is influenced by white matter injury. Of note, microglial reactivity was higher in white matter in comparison to gray matter (Mattugini et al., 2018).

The cerebellum is another remote region of the brain that can be affected by TBI. Some cortical brain injury models (FPI and CCI) result in the loss of neurons and activation of microglia and/or astrocytes in the cerebellum (Mautes et al., 1996; Park et al., 2006; Igarashi et al., 2007). Following recurrent cerebral TBI, glial response was also detected bilaterally in the brainstem and cerebellum (Bolton and Saatman, 2014).

## 8 Long-term impact of traumatic brain injury

**FIGURE 9:**
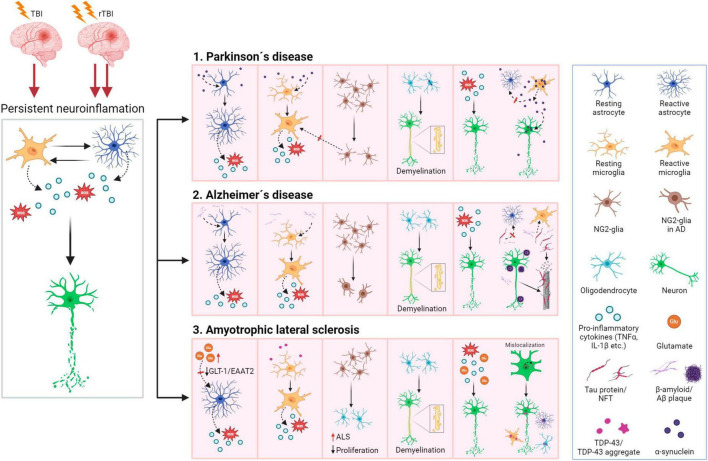
Traumatic brain injury as a risk factor contributing to neurodegenerative disease development and progression. **Left:** Persistent neuroinflammation evoked by TBI induce degenerative processes in neurons leading to their damage. Moreover, repetitive TBI has a cumulative effect on glial response which may exacerbate these processes. **Right:** Neurodegeneration itself induces glial activation and neuroinflammation, creating a vitious circle further progressing the disease. 1. Alzheimer’s disease (AD): astrocytes and microglia are activated by Aβ, and both release pro-inflammatory cytokines and ROS. Astrocytes and microglia are involved in Aβ/tau clearance, but astrocytic clearance is impaired due to loss of AQP4 polarity in the later stages of AD. The number of NG2-glia decreases, and the damage of oligodendrocytes causes demyelination of axons. 2. Parkinson’s disease (PD): microglia and astrocytes are activated by α-syn, and produce pro-inflammatory cytokines and ROS. Astrocytes and microglia can clear α-syn, but with the progression of PD, astrocyte capacity to internalize α-syn decreases, and microglia release the excess α-syn, causing more damage to the neurons. The number of NG2-glia decreases, which exacerbates neuroinflammation due to decreased suppression of microglial activation. Aggregation of α-syn in oligodendrocytes contributes to demyelination. 3. Amyotrophic lateral sclerosis (ALS): both microglia and astrocytes are activated and produce pro-inflammatory cytokines and ROS. Loss of astrocytic glutamate transports GLT-1/EAAT2 contributes to glutamate excitotoxicity. NG2-glia proliferation slows down with ALS progression and loss of oligodendrocytes induces demyelination. Additionally, TDP-43 aggregates can be found in microglia, astrocytes, and oligodendrocytes and further impair RNA stability and metabolism in the affected cells. For further details see the main text. Aβ, β-amyloid; α-syn, α-synuclein; TDP-43, transactive response [TAR] DNA binding protein 43; ROS, reactive oxygen species. Created with BioRender.com.

In view of long-term consequences, TBI is considered to be a risk factor for the development of various CNS disorders including **post-traumatic epilepsy** (PTE) (Karlander et al., 2021) and **neurodegenerative diseases**, where neuroinflammation, astrocyte activation or cerebrovascular dysfunction represent important factors contributing to the progression of the disease (Jafari et al., 2013; Tjalkens et al., 2017; Ramos-Cejudo et al., 2018; Delic et al., 2020; Brett et al., 2022; Figure 9). Interestingly, rmTBI increases the glial response which may elevate the risk of delayed disorders (Cherry et al., 2016). Indeed, studies on veterans or athletes in contact sports showed a significantly increased risk of developing sleep disorders (Leng et al., 2021; Paredes et al., 2021), neurodegenerative diseases or **motor and/or cognitive dysfunction** compared to the rest of the population (McKee et al., 2014).

### 8.1 Post-traumatic epilepsy

The link between TBI and PTE is already generally acknowledged. The chance of PTE occurrence increases with the increasing severity of injury, and the probability differs based on the type of injury, e.g., diffuse TBI (8.1%) represents a lower risk of epilepsy than focal cerebral TBI (12.9%) (Karlander et al., 2021). First seizures can occur within the first week after TBI, late epileptic seizures after weeks or months following TBI. The exact causal link between TBI and PTE development remains to be fully elucidated, but the main focus is on BBB disruption and excitotoxicity, metabolic changes, oxidative stress, and particularly on neuroinflammation (Mukherjee et al., 2020; Sharma et al., 2021). TBI is also a common predisposition to dissociative seizures, their co-occurrence being between 16 and 83% (Popkirov et al., 2018). Dissociative seizures are known as psychogenic non-epileptic seizures (PNES), or functional seizures (FS), they resemble epileptic seizures but are induced by psychological distress. However, the pathophysiology of dissociative seizures is still poorly understood (Asadi-Pooya and Farazdaghi, 2021).

### 8.2 Neurodegeneration-associated diseases

In general, TBI and neurodegenerative diseases share similar pathogenetic mechanisms, such as neuroinflammation, disrupted glutamate clearance, or mitochondrial dysfunction and production of ROS (You et al., 2023; Figure 9). Glia itself become reactive due to the neurodegeneration–reactive gliosis can be triggered by events occurring during neurodegenerative diseases, such as accumulation of β-amyloid (Aβ) in the case of AD, or cellular changes in neurons and glia during Amyotrophic lateral sclerosis (ALS) (Burda and Sofroniew, 2014). In these diseases, glia cells often play a dual role–while neuroinflammation or reversed glutamate transport can promote neurodegenerative processes, other functions of activated glia, including Aβ clearance in AD, or the production of anti-inflammatory and neurotrophic factors in ALS, can contribute to the protection of brain tissue (Lee and Landreth, 2010; Jantti et al., 2022; You et al., 2023). The relationship to TBI and the most frequent neurodegenerative diseases as well as the role of reactive glia in their pathogenesis are discussed below.

**Parkinson’s disease (PD)** is a degenerative disorder of the CNS, slowly deteriorating both motor and non-motor (e.g., cognitive or gastrointestinal) functions. A characteristic of this disease is aberrant aggregation of α-syn that disturbs dopaminergic transmission. Part of the pathology is played by neuroinflammation—aggregation of α-syn serves as stimuli for microglial activation and immune response followed by neural degeneration (Calabresi et al., 2023). Despite PD being considered as a disease of the aged brain, in cases of certain professional sportsmen, including boxers or football players, or in the case of military veterans, PD-like symptoms were also found in younger patients. Based on population-based studies, TBI is considered to be one of the risk factors for PD (Jafari et al., 2013; Camacho-Soto et al., 2017). In military veterans, the increased risk of PD following TBI was 56% (Gardner et al., 2018). However, there are too many factors, such as genetic predisposition, environment, or lifestyle that further complicate PD research within a population. To overcome some of these limitations by securing similar conditions, some studies use twins in their research. These studies in veterans from the second world war found that a person after TBI will more likely manifest a cognitive decline (Chanti-Ketterl et al., 2023) and/or develop PD in comparison with his healthy twin sibling (Goldman et al., 2006). Interestingly, White et al. (2020) suggested, that comorbidity of TBI and post-traumatic stress disorder (PTSD) further increase the risk of PD in military veterans (White et al., 2020). However, these findings need to be further confirmed by other independent studies.

Despite many population-based studies, the exact association between rTBI and an increased risk of PD is not clear. Nevertheless, due to the strong involvement of glial cells in the pathogenesis of PD, it is highly probable that shifts in receptor expression and their functions in reactive glia may be an important factor initiating or contributing to PD. Astrocytes participate in α-syn and debris clearance, but this function is impaired in PD. Additionally, α-syn activates astrocytes in the later stages of PD, which start to release pro-inflammatory factors (MHCII or IL-1β). Microglia, activated by α-syn through TLRs, release pro-inflammatory cytokines that consequently promote neuronal death. Together with astrocytes, microglia contribute also to α-syn clearance in PD, but this process inhibits microglial autophagy and α-syn begins to accumulate in the cytoplasm. The excess α-syn is then released, further exacerbating the tissue damage (Zhang et al., 2022b). There is not much information about the role of NG2-glia in PD, but in the rat model of PD, the density of NG2 cells decreased in the striatum (Nascimento et al., 2023). NG2-glia regulate neuroinflammation by suppressing microglial activation, therefore a decrease in the number of NG2-glia can lead to an exacerbated immune response (Zhang et al., 2019). Oligodendrocytes may also contain aggregates of α-syn, which is linked to myelin loss (Zhang et al., 2022b). Importantly, the glial activity and response in the PD is region-dependent (Basurco et al., 2023).

**Alzheimer’s disease (AD)** is the most common type of dementia, characterized by memory loss, ability to learn, impaired thinking and communication. According to the World Health Organization (WHO), AD represents up to 70% of all dementia cases.^1^ AD is connected with the accumulation of Aβ, which form plaques, the presence of intracellular neurofibrillary tangles (NFTs) consisting of hyperphosphorylated tau proteins, and the degeneration of cholinergic neurons. Neuroinflammation exacerbates both Aβ and tau pathologies (Kinney et al., 2018). Population-based studies have shown, that people after TBI are more likely to develop AD than people with no TBI history (Li et al., 2017; Gu et al., 2022; Mielke et al., 2022); the most likely connective factor representing the reactive pro-inflammatory micro- and astroglia in post-traumatic tissue with aberrant clearance function.

Glial cells are activated in different stages of AD, with microglia being activated first, during the period of subjective cognitive decline, and astrocytes in later stages (Nordengen et al., 2019). Astrocytes are activated by Aβ, along with released microglial inflammatory cytokines. AQP4 is thought to be important for the Aβ/tau protein clearance of interstitial solutes by astrocytes. In human patients with AD, AQP4 changes localization in comparison to healthy controls (Simon et al., 2022). In later stages of AD, the clearance is impaired due to the loss of AQP4 polarity in the astrocyte endfeet (Zeppenfeld et al., 2017; Simon et al., 2022; Pedersen et al., 2023). Aβ works as a stimulus for microglial activation and simultaneously, microglia can clear out both fibrillar and soluble forms of Aβ (Kim et al., 2018). Similarly to PD, long-term exposure of microglia to Aβ disrupts the microglial autophagy, triggers mitochondrial dysfunction, ROS production, and release of pro-inflammatory cytokines, which further supports AD progression (Litwiniuk et al., 2023). Additionally, hyperphosphorylation of tau proteins can be induced by microglial activation. Still, activated microglia can regulate the number of tau proteins through phagocytosis or by releasing tau proteins from microglia in exosomes (Uddin and Lim, 2022). NG2-glia change morphology in AD and their number is decreased in human AD (e.g., in animal APP/PS1 model of AD, NG2-glia proliferate) (Nielsen et al., 2013; Dong et al., 2018). In the early stages of AD, oligodendrocytes are already damaged, followed by myelin loss (Butt et al., 2019).

**Amyotrophic lateral sclerosis (ALS)** is a neurodegenerative disease resulting in muscle weakness, paralysis, and death, due to the degeneration of motor neurons. We can distinguish two types of ALS: familiar (approximately 10% of cases) and sporadic (You et al., 2023). To date, several contributors to neuronal damage have been identified, such as genetic mutations (SOD1, FUS, or C9ORF72) disregulating RNA metabolism, increasing oxidative stress or generating protein aggregates, including TDP-43 (transactive response [TAR] DNA binding protein 43), that can be present in neuronal/glial cytoplasm (Filipi et al., 2020). TDP-43 was shown to have multiple functions in transcriptional repression, pre-mRNA splicing and translational regulation (Sephton et al., 2011) and besides ALS tissue can be detected also in patients with chronic traumatic encephalopathy (CTE) associated with rmTBI (de Boer et al., 2020; Murray et al., 2022).

In comparison to other neurodegenerative diseases, such as AD or PD, there is not sufficient evidence, that TBI is a risk factor for ALS. However, it is believed, that reactive gliosis and inflammation (both long-term processes in TBI patients, as well as in aged brains) contribute to ALS progression (Maragakis and Rothstein, 2006). There are some population-based studies (Lian et al., 2019; Tsai et al., 2019; Filipi et al., 2020), but they have limitations (e.g,. small sample size). A further complication is the unavailability of models which would fully capture the complexity of ALS (Fisher et al., 2023). For example, one of the most frequent mutations in ALS is in Cu/Zn Superoxide dismutase 1 (SOD1) and rodents carrying the SOD1 (G93A) mutation is a commonly used model of this disease. In addition to other problems, including a decrease in the copy number of the mutation in chromosome 21 affecting ALS severity (Bonifacino et al., 2021), a recent study showed minimal disease-related changes in microglia and oligodendrocytes in SOD1 (G93A) mouse cortex during the end-stage of ALS, which contrasts with findings in patients (Filipi et al., 2023).

Amyotrophic lateral sclerosis research is primarily focused on motor neurons, but in recent years, the focus of researchers was shifted to glial cells, such as microglia and astrocytes (Neusch et al., 2007; Calafatti et al., 2023; You et al., 2023). Astrocytes in the ALS brain become reactive, they contribute to neuronal damage due to glutamate excitotoxicity caused by a loss of GLT-1/EEAT2, dysregulated release of neurotrophic factors, production of pro-inflammatory cytokines, or ROS production. Astrocytes can also contain cytoplasmic stress granules consisting of mutated TDP-43, ubiquitin, and similarly as in PD, α-syn. Microglia are activated, release pro-inflammatory cytokines (e.g., TNF-α, IL-1β) and ROS. The microglial immune response can be evoked by TDP-43 aggregates; microglia can also be more sensitive to inflammatory stimuli. TDP-43 aggregates in microglial cytoplasm in ALS patients. Very little is known about the role of NG2-glia but their proliferation and maturation into oligodendrocytes slows down with the ALS progression. Oligodendrocytes are damaged, which evokes axon demyelination of motor neurons. Similarly to astrocytes and microglia, oligodendrocytes can also contain TDP-43 aggregates (Philips and Rothstein, 2014; Filipi et al., 2020).

### 8.3 Motor and cognitive dysfunction

Traumatic brain injury-induced neuropathology significantly influences both motor and cognitive dysfunction (Gorgoraptis et al., 2019; Corrigan et al., 2023). Behavioral outcomes in various animal models that have been established to replicate diverse clinical TBI pathologies, can consequently vary. Comparing and contrasting results from different behavioral tasks within (e.g., mild vs. moderate vs. severe; acute vs. subacute vs. chronic) and between (e.g., diffuse, focal, mixed, blast) the models and pathologies is a crucial aspect in utilizing various behavioral models of TBI for a deeper understanding of its diverse aspects and subsequently for therapeutic applications (Shultz et al., 2020). Additionally, the choice of experimental species and strains is an important factor, due to differences in learning abilities, memory, attention, or responsiveness to stress and drug treatments (Fujimoto et al., 2004).

Understanding the causal mechanisms behind behavioral changes in TBI, whether in patients or animal models, is limited. Yet evidence suggests that animal models can replicate pathologies and behavioral abnormalities seen in human TBI. Here, we outline some of the common behavioral tests used for motor and cognitive assessments and model-related findings. It is important to note that protocols used in individual studies differ (even the order and number of tests can have a huge impact on the outcome), therefore the issue of standardization should be considered. Detailed information about specific behavioral tests and post-traumatic motor and cognitive deficits following different TBI models can be found in comprehensive reviews (Fujimoto et al., 2004; Shultz et al., 2020).

**Assessment of motor functions:** motor coordination tests are valuable tools for studying the effects of brain injuries on motor skills, balance and physical condition. Following TBI, motor deficits can be caused by injury-related disruption of the complex motor pathways between the cortex, sensorimotor cortex, subcortical nuclei cerebellum and brainstem (Fujimoto et al., 2004). *The Rotarod test* is commonly used to assess motor coordination and balance in rodents. In this test, the CCI model (moderate, rat) seems to impact the animal performance the most, as injury effects were detectable even 11 weeks post-TBI (Lindner et al., 1998). In comparison, midline FPI (moderate, rat) showed that injury affects performance for up to 5 days (Hamm et al., 1994). *The Beam balance test* (BBT) provides valuable insights into the impact of TBI on an animal’s ability to maintain stability and coordinate movements, which are crucial aspects of motor function. Interestingly, in this type of test, the longest impact post-TBI was found in lateral FPI (moderate, rat, the effect of injury lasted up to 1 week) (Long et al., 1996). Midline FPI (moderate, rat) affected the test performance for up to 4 days (Dixon et al., 1987) and CCI (moderate, rat) for up to 3 days (Hamm et al., 1992). *The Open field test* evaluates general locomotor activity, exploration, and anxiety-related behaviors (Fujimoto et al., 2004; McDonald et al., 2021). After CCI (mouse model), severe injury resulted to reduced locomotor activity, but not mild or moderate injury (Dixon et al., 1999; Zhao et al., 2012).

**Assessment of neurocognitive outcome:** these tests evaluate learning ability and memory. A widely used behavioral assay in TBI research is *the Morris Water Maze* (MWM) test to assess spatial learning and memory in rodents. The MWM is particularly valuable for investigating cognitive and learning deficits, working memory and retrograde/anterograde amnesia associated with TBI (Morris, 1984; Washington et al., 2012; Tucker et al., 2018). Spatial learning was impaired in the rat CCI model (moderate for more than 1 year post-TBI) (Dixon et al., 1999), and in midline FPI (moderate, rat) for up to 18 days (Yamaki et al., 1998). Mild lateral FPI (rat) did not affect learning ability, a moderate one (rat) showed an effect for up to 8 weeks (Sanders et al., 1999) and severe lateral FPI (rat) caused learning deficit for more than 1 year (Pierce et al., 1998). Effects are also severity-dependent (the mouse CCI model), with severe TBI having the worst outcome (Zhao et al., 2012). *The Barnes Maze* represents an alternative paradigm in assessing cognitive functions and allows researchers to explore the TBI impacts on a spatial navigation and memory retention in a different context than MWM (Barnes, 1979; Rosenfeld and Ferguson, 2014). In Barnes maze, rats after moderate lateral FPI developed different search patterns compared to controls (Fedor et al., 2010). Mild CCI (mouse) shows impairment after 3 months post-injury (Taib et al., 2017). Some studies reported even sex differences, where male mice displayed unique spatial memory deficits after moderate lateral FPI, but not females (Fitzgerald et al., 2022). *The Novel Object Recognition* (NOR) in TBI research serves as a behavioral assay that specifically targets declarative memory, providing valuable information on the cognitive consequences of TBI in rodent models. In the CCI mouse model, mild injury did not show any differences compared to control, but moderate and severe injury led to learning and memory impairment (Zhao et al., 2012; Fidan et al., 2016).

## 9 The role of senescent glial cells in traumatic brain injury and neurodegeneration

Senescent cells, characterized by damage and resistance to apoptosis, actively contribute to biochemical changes associated with neurodegeneration. These alterations include abnormal protein aggregation, metabolic shifts, autophagy deficits, mitochondrial dysfunction, oxidative stress, and impaired neurogenesis (Musi et al., 2018; Chapman et al., 2019; Chini et al., 2019). Senescent cells, influenced by the cell type and initiating stimulus, typically undergo a phenotypic change, with around 30–70% developing a senescence-associated secretory phenotype (SASP). SASP involves elevated, cell-dependent secretion of inflammatory cytokines such as IL-1β, IL-6, and TNF-α, chemokines, matrix metalloproteinases (MMPs), as well as proteases and growth factors. All these alterations contribute significantly to chronic inflammation and inflict damage on neighboring cells (Tchkonia et al., 2013; Baker and Petersen, 2018). Unlike neurons, glial cells exhibit a higher propensity for senescence (Salminen et al., 2011; Fielder et al., 2017; Cohen and Torres, 2019). Both astrocytes and microglia can undergo senescence, which contribute to neurodegeneration during aging or brain pathologies such as TBI, AD, and PD (Salminen et al., 2011; Baker and Petersen, 2018; Kritsilis et al., 2018).

In the context of TBI, a study using CCI in both young and aged mice revealed increased DNA damage and the expression of senescent markers in microglia within the injured brain 72 h post-injury (Ritzel et al., 2019). Senescence-associated beta-galactosidase (SA-β-Gal) positive cells, a specific marker of cellular senescence, persisted in the ipsilateral hemisphere for 1 month following TBI (Tominaga et al., 2019; Schwab et al., 2021). Elevated SA-β-Gal was observed throughout the cortex, hippocampus, and thalamus at 1 day and 1 month following a single or repeated moderate bTBI in rats (Arun et al., 2020). Moreover, SA-β-gal activity remained elevated for over 2 weeks post-TBI, accompanied by an increased expression of p16INK4a, a cyclin-dependent kinase inhibitor associated with senescence, specifically noted in astrocytes following TBI (LaPak and Burd, 2014; Tominaga et al., 2019).

Additionally, postmortem brains of professional athletes with multiple mTBI similarly showed increased DNA damage, senescence marker expression, and the presence of SASP factors (Schwab et al., 2019). Moreover, rmTBI in mice exhibited elevated DNA damage markers at 24 h and increased expression of p21, p53, and SASP, particularly IL-1β, in the injured cortex 7 dpi (Schwab et al., 2021). However, the administration of ABT263, the senolytic drug, 1 week after rTBI significantly enhanced performance in the MWM (Schwab et al., 2022).

Several important conclusions emerged from a study by Wang J. et al. (2023). Following TBI, their findings supported the long-term neurodegeneration in the cortex, hippocampus, dentate gyrus, and thalamus, associated with a strong neuroinflammatory response, white matter damage, and a significant deficit in cognitive function and depressive-like behavior (Wang J. et al., 2023). These results are in line with human research showing that white matter damage and neuroinflammatory events in the brain can last for many years following TBI (Johnson et al., 2013b). Furthermore, nearly 50% of hospitalized TBI patients have impairments from their injuries after a year, including depression, anxiety, and cognitive impairment (Johnson et al., 2013b). Secondly, an investigation by Wang J. et al. (2023) revealed widespread occurrence of senescent cells in various brain regions persisting for weeks to several months following TBI. Through double immunohistochemistry, it was observed that these senescent cells included both astrocytes and microglia. This observation aligns with prior studies that identified senescent astrocytes and microglia in the injured cortex shortly after TBI (Ritzel et al., 2019; Tominaga et al., 2019). Recent findings indicate that senescent astrocytes, displaying reduced neuroprotective functions due to glutamate transporter down-regulation, may contribute to prolonged neurodegeneration post-TBI (Limbad et al., 2020).

Senolytic therapy, eliminating senescent astrocytes and microglia, resulted in reduced neurodegeneration and increased neuron numbers in multiple brain regions in animals with TBI (Wang J. et al., 2023). Senescent cells also release SASP factors, promoting chronic neuroinflammation and causing damage to neighboring cells. Wang J. et al. (2023) also showed that proinflammatory SASP factors, IL-1β and IL-6 were significantly elevated in the ipsilateral cortex at 4 months post-TBI and a senolytic therapy with dasatinib and quercetin (D + Q) treatment markedly reduced this increase, indicating a substantial reduction in the long-term inflammatory burden (Wang J. et al., 2023). Persistent inflammation after TBI is linked to unfavorable outcomes (Kumar et al., 2015). D + Q therapy not only lowered the senescent cell burden but also mitigated chronic neuroinflammation, aligning with the potential benefits of targeting IL-1β and IL-6, known to improve cognitive function after TBI (Clausen et al., 2011; Flygt et al., 2018). The potential involvement of senescent cells in prolonged functional deterioration post-TBI is underscored by the effectiveness of delayed, intermittent D + Q senolytic treatment initiated 1 month after TBI. This intervention notably eradicated senescent cells and resulted in a significant amelioration of both cognitive impairment and depressive-like behavior in TBI mice at the 4-month post-injury interval. This extension of the therapeutic window, beyond the conventional acute treatment timeframe, holds clinical significance by offering potential interventions for patients well into the chronic phase of TBI, where therapeutic options are currently scarce (Wang J. et al., 2023). For further information about senolytic and senomorphic therapy see chapter “TBI diagnostics and treatment.”

## 10 Traumatic brain injury in military and veterans and special features of mild blast injury

Soldiers and war veterans are a group of TBI patients providing valuable data for population-based studies of TBI—their medical history is usually well documented while being at high risk of TBI, such as BI, bullet penetration, falls, or head impacts. According to the Defense and Veterans Brain Injury Center (DVBIC), approximately 82% of reported TBI sustained by members of the U.S. Armed Forces between 2000 and 2023 was classified as mTBI.^2^ TBI affecting military personnel was linked or correlated with visual dysfunctions, sleep disorders, development of psychiatric sequelae including depression and PTSD, and higher risk of neurodegenerative disorders (McKee and Robinson, 2014; Hussain et al., 2021; Leng et al., 2021; Kong et al., 2022).

The most common TBI affecting soldiers is mild bTBI after exposure to explosives and their overpressure wave (McKee and Robinson, 2014; Hernandez et al., 2018). However, soldiers are not the only ones affected by bTBI, the civilian population can suffer bTBI due to terrorist bomb attacks or presence in an active war zone. Overpressure waves cause compression of brain tissue as well as the movement of the brain in the skull, causing more damage by impact (Bryden et al., 2019). These events evoke hemorrhaging, edema, axonal injury, or neuroinflammation (McKee and Robinson, 2014).

Blast TBI exhibit common as well as unique features compared to concussive-type TBI, where there is evidence of astrogliosis, maladaptive inflammatory response and BBB breakdown (Rubovitch et al., 2011; Wilk et al., 2012). Close analysis has shown the induction of microvascular and axonal damage and neuroinflammation in the cortex, striatum and hippocampus. These changes correlate with deficits in synaptic function and hippocampal plasticity. Biochemical analysis revealed overactivated calpain, indicating disrupted Ca^2+^ homeostasis after mild bTBI (Hernandez et al., 2018).

Logsdon et al. (2018), in agreement with other studies confirmed that a single blast exposure rapidly and temporarily disrupts the BBB, allowing abnormal entry of sucrose and albumin traces into the various brain regions. BBB integrity is restored for albumin but not sucrose by 24 h (Logsdon et al., 2014, 2018; Huber et al., 2016; Mishra et al., 2016). Following two consecutive mild blast exposures cause the BBB to reopen to albumin at 72 h. Tight junction disintegration aligns with the second phase of BBB disruption. These results indicate immediate and prolonged brain permeability to small molecules, coupled with delayed, region-specific permeability to larger molecules (Goodrich et al., 2016). Despite the prevalent role of astrocytes in TBI, their contribution to mild bTBI remains unclear. In blast-exposed swine hippocampus, elevated expression of inflammatory proteins has been found (Goodrich et al., 2016), supporting the idea that blast-induced inflammation may involve astrocytic activation. Astrogliosis observed in animal BI and CHI models (Kernie et al., 2001) also appears in post-mortem brains of blast-exposed soldiers, particularly evident in the hippocampus. Goodrich et al. (2016) on the model of Yorkshire pigs showed activation of astrocytes 2–4 weeks post single blast exposure (Goodrich et al., 2016). In animals exposed to double or triple blasts with longer survival times (6–8 months), there is a 20–30% increase in activated astrocytes in the dentate hilus and molecular layer compared to single blast exposure. Elevated astrocyte density, noted in CA1, may respond to observed neuronal injury. In the hilus, where significant neuronal injury was observed in the single blast-exposed group, but an increased astrocyte number suggests an early involvement in the hippocampal injury process. Proteomic analysis confirms a rapid elevation in GFAP levels, supporting early astrocyte activation post-blast (Ahmed et al., 2012). On the other hand, microglial activation is not so widespread (Goodrich et al., 2016). Activated microglia, mainly in white matter regions like the corpus callosum, were prominent in animals with double and triple blast exposure. However, despite their presence, there was no detectable axonal degeneration observed through Aβ immunostaining in the same areas, while in post-mortem brains of blast-exposed soldiers, activated microglia were associated with patches of Aβ injured axons. The precise role of activated microglia in blast mTBI in white matter tracts thus remains unclear. More findings regarding glia in rodent model of repetitive bTBI are available in Table 1.

**TABLE 1:** Overview of rTBI experimental models with a focus on glial cells.

TBI model	References	Astrocytes	Microglia	NG2-glia	Oligoden-drocytes	Animal	Age	Sex	Anesthesia	Severity	Repetition	Impact total
WD (Shohami)	Briggs et al., 2016	↑GFAP in Ot, Cc	↑Iba1 (Ot, Cc)	–	↓MBP, ↑olig2^+^ cells (Ot*, Cc)	Mouse C57BL/6	7–8 weeks	M	Isoflurane	Mild	24 h (5x), 48 h rest	30
WD (Marmarou)	Shandra et al., 2019	↑GFAP (7 dpi, Cor-GM)	–	–	–	Mouse C57BL/6 or Aldh1l1-eGFP//FVB/N mouse	12–16 weeks	M, F	Isoflurane	Mild	45 min interval	3
WD (Marmarou)	Xu et al., 2016	↑GFAP in Ot	Activated Iba1^+^ cells (Sc, Cst, Cc, Cb-WM); M: deramified, large, often clustered	–	–	Mouse C57BL/6	5–6 weeks	M	Isoflurane	Mild	D 0, 1, 3, 7 (1x/3x per D)	4/12
WD (Feeney)	Allen et al., 2000	↑GFAP in ipsi + contra Cor, Hi, GFAP overlap w/Hsp27	–	–	–	Rat Sprague-Dawley	Adult	M	Sodium pentobarbital	Mild + Severe	72 h (3x), 3 or 5 days after severe TBI	3 or 4
CCI	Yu et al., 2017	↑GFAP area in Cc, Cor	↓CD11b area + density (Cc, Cor)	No change (NG2) in Cc, Cor	No change (MOG) in Cc, Cor	Mouse C57BL/6	8 weeks	M	Isoflurane	Mild	24 h interval	5
CCI-CHI	Bolton and Saatman, 2014	↑GFAP^+^ cells, GFAP in Ent, Cb, Bs	Morph. changes (Iba1) in Ent; MHCII no change	–	–	Mouse C57BL/6	2–3 months	M	Isoflurane	Mild	24/48*h interval	5
CCI-CHI	Shitaka et al., 2011	–	↓Iba1; persistent in Cc, transient in gray matter; M: H, B, A	–	–	Mouse C57BL/6	2–3 months	M	Isoflurane	Mild	24 h interval	2
CCI-CHI	Uryu et al., 2002	Reactive gliosis in Cor, Hi (2 dpi), Cor (9 wpi)	–	–	–	Mouse WT + Tg2576	9 months	M, F	Sodium pentobarbital	Mild	24 h interval	2
CCI-CHI	Bennett et al., 2012	–	Iba1^+^ cells in Cc	–	–	Mouse C57BL/6J	6–8 weeks	M	Isoflurane	Mild	24 h	2
CCI-CHI	Luo et al., 2014	↑GFAP in ipsi Cor, Cc, Hi (CA3), cumulative effect of rTBI; M: H, large cell body, densely labeled processes	–	–	–	Mouse C57BL/6J + GFAP-luc	2–3 months	M, F	Isoflurane	Mild	24 ± 1 h	2, 3 or 5
Lateral FPI	Aungst et al., 2014	–	Activated microglia (Iba1) in Cor, Hi; M: H, B	–	–	Rat Sprague-Dawley	10–12 weeks	M	Isoflurane	Mild	D 0, 2, 4	3
Lateral FPI	Shultz et al., 2013	↑GFAP, reactive astrogliosis (injury area); M: long hypertrophic processes	↑ED1 (CD68), ↑ED1^+^ cells in Cor (to deeper layers); M: rounded	–	–	Rat Long-Evans hooded	N/A	M	Isoflurane	Mild	D 1, 6, 11	3
Lateral FPI	Fronczak et al., 2022	↑GFAP in subCor (WM), Th (2 impacts)	↑Iba1 in Cor, Th, subCor (WM), but not in Hi (2 impacts); M: rod shape (Cor)	–	–	Rat Sprague-Dawley	8–12 weeks	M	Isoflurane	Mild	24 h interval	2 or 4
BI	Campos-Pires et al., 2023	GFAP^+^ area no change (Cor, subCor), 24 h/5 dpi	–	–	–	Rat Sprague-Dawley	10–12 weeks	M	Propofol	Mild	7.0 ± 0.2 min	3
BI	Bugay et al., 2020	↑GFAP (Hi–DG; but not CA1); ↑GFAP in rostral*, middle, caudal region (WB)	↑Iba1 in Hi (DG, CA1*)	–	–	Mouse C57BL/6J	10 weeks	M	Ketamine + Dexmedeto-midine	Mild	24 h interval	3
BI	Dickerson et al., 2020	↑GFAP and reactive astrocytes in Th (CM); M: increased soma	↑Iba1 density in Th (CM), ↑Iba1 area fraction in Th (VL); M: increased soma (CM, but not VL)	–	–	Rat Sprague-Dawley	N/A	M	Isoflurane	Mild	1 h interval	3
BI	Kumari et al., 2023	↑GFAP^+^ cells* (Hi)	–	–	–	Rat Sprague-Dawley	10–12 weeks	F	Isoflurane	Mild	0 min, 30 min, 24 h	3

↑Upregulation/increase. ↓Downregulation/decrease.

*Not significant. Morphology (M): H, hypertrophic; B, bushy; A, ameboid. Brain regions: Cst, corticospinal tract; Sc, superior colliculus, Ot, optic tract; Ent, entorhinal cortex; Cb, cerebellum; Bs, brainstem; Cor, cortex; Hi, hippocampus; DG, dentate gyrus; Cc, corpus callosum; subCor, subcortical regions (amygdala, ventromedial hypothalamus, centromedial thalamic nucleus, ventromedial/ventrolateral thalamic nucleus, ventral posteromedial thalamic nucleus, laterodorsal thalamic nucleus, ventrolateral, and medial habenular nucleus and hippocampus); Th, thalamus; CM, central medial nuclei; VL, ventrolateral nuclei. Other: Sex: M, male; F, female. MOG, myelin oligodendrocyte glycoprotein; MBP, myelin basic protein; WM, white matter; GM, gray matter; D, day; ipsi, ipsilateral; contra, contralateral; WB, western blot; N/A, not available; CD11b and CD68/ED1, cluster of differentiation/endothelin 1; produced by microglia and macrophages; GFAP, glial fibrillary acidic protein; Iba1, ionized calcium binding adaptor molecule 1; dpi, days post-injury; wpi, weeks post-injury; protein^+^, protein-positive.

Recent studies delve deeper into the impact of mild blasts on cognitive, metabolic, and behavioral functions. Kumari et al. (2023) specifically explored hippocampal metabolic changes after mild, moderate and rmTBI in rats (Kumari et al., 2023). Significant alterations in N-Acetylaspartic acid (NAA), acetate, PC/choline, phosphoethanolamine (PE), ethanolamine, glutamate, and creatine concentrations were noted compared to Sham only in the rmTBI group. These metabolic shifts may affect biochemical processes and metabolic-dependent mechanisms such as the epigenome, highlighting the intricate link between metabolism and epigenetics (Kumari et al., 2023). A study by Kumari et al. (2023) also showed an Alanine (Ala) reduction in the hippocampus 24 h post-mild, moderate, and repeated bTBI, which aligns with another study by Norris et al. (2023). This study, quantifying acute changes after bTBI in the metabolism of free amino acids as molecular precursors, neurotransmitters and metabolic regulators in rats, found alterations in Arg and Ala pathways linked to oxidative stress following bTBI (Norris et al., 2023). Decreased Arg may elevate oxidative stress via NOS and contribute to pro-inflammatory cascades (Mader and Czorlich, 2022). Administering Arg post-TBI showed neuroprotective effects, while the NOS inhibitor L-NAME improved the BBB function and sensorimotor impairment post-blast exposure (Logsdon et al., 2020). Modulating the NO/Arg pathway may thus have therapeutic potential, warranting further investigation into acute Arg changes in various tissues.

Animal models of bTBI may be restricted due to interspecies variations in the neuropathophysiology between the animal and human brain. Using 2D or 3D human cell cultures for *in vitro* modeling also has its limitations (Fang and Eglen, 2017; Qian et al., 2019). Snapper et al. (2023) introduced a new *in vitro* model based on a bioengineered 3D brain-like culture system that can mimic the human pathology fairly well (Snapper et al., 2023). Using this novel bTBI model they revealed increased axonal varicosities following primary bTBI, persisting for a week with a peak at 72 h. These varicosities, indicative of traumatic axonal injury, show the accumulation of amyloid precursor protein (APP), suggesting a blast-induced deficit in axonal transport. The study also found elevated NF-H and UCH-L1 (Ubiquitin carboxy-terminal hydrolase L1, known neuronal damage markers) at 6 h, S100B (astrocytic marker) at 24 and 48 h post-blast, indicating sequential damage. GFAP didn’t increase, possibly due to elevated pre-injury levels. This novel *in vitro* culture system without a BBB may allow controlled screening for new biomarkers that are pivotal in diagnose blast-induced mTBI (Snapper et al., 2023).

## 11 Factors affecting traumatic brain injury outcome in experiments

Traumatic brain injury is a complex process and due to its intricacy, creating a model that closely mimics human pathology is difficult. As many previous studies have shown, there are differences within TBI models, including their impact on glial cells and different reported results depending on the severity and location of injury as well as on model accuracy, reproducibility and repeatability. However, the type of TBI model is not the only aspect which is necessary to be considered—factors, such as age and sex of laboratory animals are also essential for the glial response. In this section, we will summarize some of these factors and their impact on glial cells.

### 11.1 Age and the impact of aging on traumatic brain injury

The age of an experimental animal is a crucial concern when designing experiments. Compared to the youthful brain, glial cells in the aged brain exhibit changes in shape and protein expression (Alibhai et al., 2018). Based on the genomic analysis of ischemic tissue, Androvic et al. (2020) unveiled a significant overlap in the inflammatory response, cell-cell interactions, and cell cycle progression between young adult, and aged animals (Androvic et al., 2020). Animals commonly used in research experiments are frequently in the prime of their productive adult lives. Nevertheless, it is important to note that the aging brain is more susceptible, and the elderly are at an elevated risk of TBI due to pre-existing medical conditions, a heightened likelihood of falls, natural anatomical changes, and other factors, as discussed by (Yee and Jain, 2023). Another crucial factor is the fluctuations in hormonal levels during aging, as they have a significant impact on the glial response (Arevalo et al., 2010). Since the female hormone cycle and/or menopause may have an impact on TBI outcomes, the inclusion of females in studies of the aging effect on TBI results is crucial.

During aging, an increased expression was detected in some proteins that were shown to be involved in the tissue response to TBI, such as GFAP, Vim, and AQP4 (Nichols et al., 1993; Porchet et al., 2003). Moreover, astrocytic subpopulations that differ from those in young tissue in morphology, function, or membrane properties, were identified during aging (VanGuilder et al., 2011; Matias et al., 2019; Verkerke et al., 2021). Based on RNA sequencing, Clarke et al. (2018) found that due to aging, astrocytes change their transcriptome and become reactive in a region-dependent manner. In response to the neuroinflammation promoted by microglia, the number of reactive astrocytes is significantly higher in aged tissue than in young brains (Clarke et al., 2018). During aging, microglia undergo morphological and functional alterations and subsequently become senescent/dystrophic (Streit et al., 2004). Aging is also associated with microglial priming which results in higher sensitivity to stimuli and exaggerated microglial responses (Henry et al., 2009).

It was demonstrated that a cognitive decline in aged patients, besides other morphological brain changes, is also associated with an overall loss of white matter (Chopra et al., 2018). The most impacted structures are the anterior-posterior gradient and the prefrontal cortex, and the anterior corpus callosum (Head et al., 2004; Bennett et al., 2010). The white matter damage seems to be promoted by persistent NF-κB signaling, which is a pathway also inducing the senescence of oligodendrocytes (Schlett et al., 2023). Waller et al. (2019) investigated the microglial populations present in deep subcortical lesions (DSCLs) of the brain, which are present in approximately 60% of the population over 65 years. DSCL is a white matter lesion linked with myelin loss, BBB dysfunction, and gliosis. In human autopsy tissue, a significant decrease of microglia marker Iba1 and an increase of CD68 expressions in the DSCL group as well as shifted morphology of CD68-positive (CD68^+^) cells from ramified to more rounded cells were found. Of note, some CD68^+^ cells did not express Iba1, indicating the presence of microglial subpopulations. The authors also found that myelin integrity in DSCL was reduced. In contrast, another type of white matter lesions presented in aged tissue, periventricular lesions (PVLs), contained more activated microglia with a ramified phenotype. In this phenotype, a higher MHCII expression was detected, as well as increased expression of B7-2 and CD40 (both co-stimulatory molecules of MHCII) than in the DSCL microglia (Waller et al., 2019). PVL is also a more microglia proliferation supporting environment than DSCL (Simpson et al., 2007). Additionally, age-related impairment of progenitor recruitment and the consequent differentiation into oligodendrocytes causes a decrease in remyelination efficiency in the elderly (Sim et al., 2002).

Following CCI, age-related significant differences in microglial activation were detected (Kumar et al., 2013). This study showed increased expression levels of genes such as IL-1β, CD86, and TNF-α, upregulation of MHCII mRNA and upregulated expression levels of MHCII, as well as a higher number of activated microglia in the cortex, hippocampus, and thalamus. Moreover, 2 different microglial phenotypes were observed. The authors also revealed that the volume of lesions in the aged brain is higher, which is combined with a significantly increased neuronal loss in the aged group, in comparison with the young group (Kumar et al., 2013). Compared to young adult tissue, the astroglial and microglial reaction after CCI was delayed in the hippocampus of the aged brain; a similar response of astrocytes was detected in the cortex (Sandhir et al., 2008; Early et al., 2020). Furthermore, the hippocampus appeared to be more vulnerable to TBI-induced morphological changes of reactive astrocytes (Early et al., 2020).

Besides astrocytes and microglia, the function of other glial cells during aging is also altered. NG2 cells in gray matter differentiate into oligodendrocytes faster in young mice than in older mice (Hill et al., 2014). Viney et al. (2022) used the THY-Tau22 tauopathy mouse model to show the spread of phosphorylated tau from neurons to oligodendrocytes in the hippocampus of aged mice (Viney et al., 2022).

These alterations may have a great impact on tissue damage and recovery of the aged brain after TBI, as well as higher levels of neurodegeneration in the elderly. Unfortunately, TBI models do not reproduce responses in aging brains in full detail, since aged experimental animals lack the comorbidities usually found in the elderly, such as diabetes, hypertension, and cardiovascular diseases.

### 11.2 Sex differences

Based on epidemiological studies, the incidence of TBI is higher in men than in women (Faul and Coronado, 2015; Peeters et al., 2015; Capizzi et al., 2020; Brazinova et al., 2021). Unfortunately, the study sample based on sex is not usually equally distributed (Bodnar et al., 2019; Gupte et al., 2019; Biegon, 2021), therefore, the results of some studies may be misinterpreted. The levels of hormones progesterone and estrogens, especially estradiol, seem to be an important factor in TBI outcome (Roof et al., 1994; Bramlett and Dietrich, 2001). Specifically, 17β-estradiol modulates the adult microglial phenotype and decreases neuroinflammation after TBI (Wang et al., 2021). The ability of these hormones is in tight cooperation with astrocytes and microglia (Johann and Beyer, 2013). Menzies et al. (2011) demonstrated that progesterone can shift the activation type of the macrophages by the selective regulation of macrophage gene expression (Menzies et al., 2011).

In some studies, with a focus on the sex of the experimental animals, differences in glial cell response were observed (Acaz-Fonseca et al., 2015; Villapol et al., 2017). For example, in the CCI model, males displayed more rapid astrogliosis and a larger increase in lesion volume in comparison to females (Villapol et al., 2017). Moreover, the study also revealed differences in microglial activation, morphology, and phenotype diversity, as well as in the inflammatory response and mRNA levels, with a greater extent of reactive microgliosis found in male rather than female tissue post-TBI. The differences in neuroinflammatory responses were also observed in the cortical stab injury model. Interestingly, the number of neurons in the lesion site was higher in males, but no difference in the number of astrocytes between males and females was detected. Male samples showed a higher percentage of an astrocytic subpopulation expressing chemokine (C-C motif) ligand 2, which is an important mediator of neuroinflammation (Acaz-Fonseca et al., 2015). In the WD model, only mild differences in glial response between sexes, even in rTBI were observed (Shandra et al., 2019). Males and females also exhibited different results in behavioral testing post-TBI in the lateral FPI model, with females expressing greater sensitivity to sensory stimuli, such as light or noise, than males (Hoffman et al., 2020).

### 11.3 Chemicals used for anesthesia

Anesthesia is widely used in experiments to prevent animal suffering during the induction of TBI. In general, anesthesia, especially long-term exposure can result in brain damage, specifically the induction of the mitochondrial apoptosis, which evokes neuronal death (Mesa Suarez et al., 2016; Wu et al., 2019). Furthermore, the following studies revealed the effect of different chemicals used for anesthesia on the reaction of various glial cells. Drugs used for induction of general anesthesia, such as isoflurane, suppress LPS-induced expression of microglial pro-inflammatory cytokine IL-1β in mouse brains (Tanaka et al., 2013). On the other hand, prolonged anesthesia using sevoflurane induces neuroinflammation by triggering the NF-κB pathway, activates microglia, and alters their morphology in the hippocampus of aged rats (Xu et al., 2023). According to Brambrink et al. (2012), exposure to isoflurane for 5 h causes apoptosis of oligodendrocytes (estimated at 6.3% of the total oligodendrocyte population) in neonatal primate brains (Brambrink et al., 2012). Isoflurane also inhibits Na^+^/K^+^-ATPase, specifically causing a decrease in the activity of astrocytic and neural isoform of Na^+^/K^+^-ATPase (approximately 25% decrease after 1-h exposure) and therefore disrupting ion homeostasis (Reiffurth et al., 2023). Sun et al. (2019) compared isoflurane and ketamine anesthesia using a mouse model with a cranial window. Isoflurane caused significantly elongated microglial processes in both acute (1–2 days post-surgery) and chronic experiments (4 months after surgery) and a faster reaction of microglia to photodamage in chronic trials. Ketamin only increased the number of microglial branching in acute experiments (Sun et al., 2019). Furthermore, isoflurane decreases the expression of α-tubulin and GFAP directly during anesthesia and even 2 days following 4 h of exposure *in vitro* (Culley et al., 2013).

### 11.4 Frequency of traumatic brain injury

Multiple mild head injuries are risks associated with military service or contact sports, such as boxing. The importance of investigating rmTBI is high, because of the link to the development of CTE or a higher risk of dementia/AD (Uryu et al., 2002; McKee et al., 2009, 2016). Cherry et al. (2016) studied the post-mortem brains of American footballers and discovered an altered microglial phenotype, and increased density of DC68^+^ cells. The authors then correlated the increased neuroinflammation with longer exposure to rmTBI. These results propose the theory that cumulative damage results in a stronger glial response (Cherry et al., 2016). Amplification of glial activation was also shown in the CCI-CHI model with a midline impact resulting in bilateral injury; TBI repeated after 24 h exhibited a significantly increased microglial response than single impact, but prolonging intervals between TBI to 48 h evoked similar results to the single TBI (Bolton and Saatman, 2014). Similarly, results in lateral CCI-CHI repeated after 24 h revealed that rmTBI–affected brain tissue evinced increased microglial activation in comparison to single TBI. The level of the microglial response in the observed brain regions was variable in time, with the largest microglial response to rmTBI at 4 dpi in the hippocampus, 7 dpi in the cortex, and 28 dpi in the thalamus (Shitaka et al., 2011).

Xu et al. (2016) observed activated deramified microglia (Iba1^+^ cells, many of them also CD68^+^) in the superior colliculus, corticospinal tract, corpus callosum, and part of the cerebellar white matter in rmTBI using the Marmarou WD model. Additionally, phagocytic reactive microglia colocalized with injured axons in the optic nerves, optic tract, corticospinal tract, and cerebellar lobule (Xu et al., 2016). Briggs et al. (2016) reported astrocytic and microglial reactivity in the corpus callosum and optic tract, white matter thinning of the corpus callosum, and myelin loss in the WD model of injury. Their data also suggest that rmTBI has an accumulative effect. However, their conclusion could be misleading, as instead of comparing single-impact mTBI with rmTBI, the authors only compared rmTBI animals with control animals anesthetized with isoflurane (Briggs et al., 2016). On the other hand, the population of NG2-glia in CCI rmTBI was not affected in the corpus callosum or cortex; a significant change in myelination was not detected either (Yu et al., 2017). The rmTBI also exacerbates the astroglial response in the CCI-CHI model if repeated after 24 h (Uryu et al., 2002; Bolton and Saatman, 2014). However, in the WD model of rmTBI, Shandra et al. (2019) did not observe an induction of GFAP^+^ astrocyte proliferation (Shandra et al., 2019). We have compiled these research findings concerning glial cells in experimental models of rmTBI, based on the results discussed in this article in Table 1.

## 12 Genomic methods in traumatic brain injury research

An important aspect of TBI research involves the development of novel methods, their improvement, and integration with established ones. Advanced methods have emerged in neuroscience in recent years, specifically focusing on the application of cutting-edge procedures of single-cell transcriptomics.

For single-cell transcriptomics, two basic methods are used: single-cell RNA sequencing (scRNA-seq) and single-nucleus RNA sequencing (snRNA-seq). The samples for scRNA-seq must be fresh and require tissue disintegration. Alternatively, samples for snRNA-seq can be frozen (an advantage when using samples from patients) or fixed. Additionally, snRNA-seq is more suitable for fragile cells (e.g., neurons), which are difficult to dissociate intact. However, the disadvantage of snRNA-seq is analysis predominantly focused on nuclear transcripts. Both scRNA- and snRNA-seq methods have their limits, for example they lack spatial information (Piwecka et al., 2023). To overcome the limitations and biases of sc/snRNA-seq, other studies use some of the following strategies: multimodal analysis (more than one molecular feature is analyzed, integrating data from transcriptome with epigenome or proteome from the same cell), combination with physiology or morphology analysis (incorporation with electrophysiological recordings, e.g., scRNA-seq and calcium imaging), spatial transcriptomics (RNA analysis while spatial information is preserved, mapping the positions of cells using *in situ* hybridization), or perturbation experiments (integration of genome editing for loss/gain functional studies) (Mayer et al., 2019; Armand et al., 2021).

scRNA-seq allows to reveal transcriptomic shifts or cell-to-cell interactions (by mapping gene co-expression). For example, in the model of lateral FPI, injury enhanced interaction between astrocytes and neurons, as well as between microglia and oligodendrocytes, while the interaction of microglia and oligodendrocytes with neurons was decreased in comparison with the controls (Arneson et al., 2018). These observations suggested TBI-dependent reorganization processes. The most enriched DEGs (differentially expressed genes) after TBI were those involved in metabolic depression and calcium/calmodulin pathway in astrocytes, inflammation in microglia, myelination and oligodendrocyte differentiation in oligodendrocytes and myelination and immune response in OPCs. The most significantly differentially expressed cell-type specific genes were *p2ry12* (gene of purinergic receptor, role in microglial activation) in microglia, and *Trf* (gene of transferrin receptor) in oligodendrocytes (Arneson et al., 2018).

Arneson et al. (2022) identified a new treatment target, humanin and its gene *mt-Rnr2*, based on spatiotemporal alterations in the lateral FPI model. Expression levels of several DEGs which were elevated post-injury, decreased after humanin administration. Additionally, the treated animals showed improvement in cognitive ability. The authors also mapped TBI-evoked region-dependent changes in glial cells in the hippocampus and frontal cortex during the acute and subacute phases of TBI. In the hippocampus, the genes involved in oxidative phosphorylation and ETC were downregulated in astrocytes and oligodendrocytes (acute phase), while in microglia, TBI induced upregulation of metabolic pathways (subacute phase) (Arneson et al., 2022).

Garza et al. (2023) used snRNA-seq to analyze human samples after severe TBI. Injury causes transcriptional changes in oligodendrocytes, triggering an immune reaction and interferon response (via STAT1/2) that activates MHC-related genes. Similarly, immune processes can also be detected in OPCs. In addition, transcription of several endogenous retroviruses was activated in oligodendrocytes and OPCs, and some level of activation was detected in microglia following TBI. This retroviral activation could potentially lead to the activation of immune pathways (Garza et al., 2023).

Microglia and astrocytes are the most affected cells in the subacute phase in the lateral FPI model. During this phase, several signaling pathways were enhanced including CCL (CC chemokine ligand, indicates neuroinflammation) and non-canonical neurotrophic factor signaling. Non-canonical neurotropic factors MDK (Midkine), PTN (Pleiotrophin), and PSAP (Prosaposin) were upregulated in astrocytes and microglia. Based on *in vitro* testing, astrocytic expression of MDK, PTN, and PSAP is enhanced by activated microglia. LPS induction directly evoked only upregulation of PSAP in BV2 microglia (Qiu et al., 2023).

Data from sequencing can be processed into a library or interactive atlas (Network, 2021; Zhang et al., 2023). Such atlases are available, for example on the online platform Allen Brain Cell Atlas, created from scRNA-seq and spatial transcriptomic datasets from the whole mouse brain (Yao et al., 2023). Hence, these datasets and atlases present opportunities to uncover connections that were challenging to discern across a diverse array of fields. Sc/snRNA-seqs are also very useful in the investigation of brain tumors, neurodegeneration, or psychiatric disorders (Antunes and Martins-de-Souza, 2023; Pozojevic and Spielmann, 2023; Walter et al., 2023). Since even small changes in the transcriptome can be detected, it can help to identify the involvement of different cellular subtypes in diseases or outline time-dependent changes which would be otherwise undetectable. The application of single cell/single nucleus methods also have a significant impact on clinical research, showing the possibility of new treatment targets as well as tools for effective diagnostics.

## 13 Current challenges and future directions

The field of TBI research is highly dynamic, and presents many challenges, which can be overcome only with newly developed and improved techniques and analytic methods. Here, we list some of the most important items that should be considered in future research:

### 13.1 Heterogeneity of traumatic brain injury outcome in experimental models and the model limitations

Traumatic brain injury animal models are useful and still irreplaceable tools in brain injury research. However, TBI is not simple to imitate, the observations in patients are complex, and to date, no model can contain all of them. The key to success is to carefully design the experiment and determine which aspect of secondary injury we would like to focus on. The absence of comorbidities, which are present in older patients, the impact of hormones and their fluctuation, the variability of glial morphology, and expression profiles are all complications that can arise when designing an experiment using animal models. In general, many studies appear to focus on the acute phase of TBI, using mostly males to avoid variability in experimental groups, and mainly young adult animals, despite the high occurrence of TBI in the elderly and the higher possibility of worse outcomes post-TBI. We also noted a lack of articles with a focus on NG2-glia and oligodendrocyte functions in rmTBI.

### 13.2 Long-term consequences of traumatic brain injury

As aforementioned, many studies appear to focus on the acute phase of TBI. However, TBI should be handled more as a “chronic disease.” Various studies using data from both patients and animal models show long-term consequences of TBI, including physiological and/or cognitive impact. For example, a study from 2022 shows that up to 56% of patients with mTBI do not fully recover after 6 months (Madhok et al., 2022). Additionally, TBI is linked to several neurodegenerative diseases, such as AD, PD, or CTE, which was proved by population-based studies and confirmed by following animal experiments. Therefore, an effective and early treatment of TBI, based on understanding the chronic effects, could potentially shorten the recovery period, and limit lifelong consequences, as well as delay the onset of neurodegenerative diseases or alleviate their symptoms.

### 13.3 Traumatic brain injury diagnostics and treatment

For TBI patients, it is crucial to be properly diagnosed and treated as soon as possible. However, patients are still treated based on the severity of TBI, while neglecting the type of TBI and its pathologies. To distinguish them requires specific biomarkers, which would be specific to TBI, easy and fast to test, and become a stepping stone to accurate diagnosis (e.g., assist the severity or phase of TBI). Data available from biomarker testing would also help in research for more precise analysis. In 2023, U.S. Food and Drug Administration (FDA) approved the use of enzyme-linked immunosorbent assay testing GFAP and UCH-L1 presence in blood, to determine the need for CT or MRI scan.^3^ However, the need for improved diagnostic tools is still actual.

The existing commonly used therapy of TBI is causal but mostly unspecific (for example treatment of brain edema using mannitol, corticoids and diuretics) and does not aim directly at molecular and cellular mechanisms of the brain injury. It is therefore crucial to develop more sophisticated up-to-date approaches, similar to the glia-oriented search for therapeutical targets in treatment of stroke (Hernandez et al., 2021).

In TBI, glia-oriented **senolytic therapies** seem to be very promising not only in animal experiments but even in clinical trials. Senolytic and senomorphic therapies aim to improve cognitive outcomes by removing or modifying senescent cells. Efforts to identify senotherapeutic agents, have led to the discovery of various classes of senolytics, including kinase inhibitors, Bcl-2 family inhibitors, natural compounds, p53 inhibitors, heat shock protein 90 inhibitors, and histone deacetylase inhibitors (HDAC). Given the potential acceleration of aging after TBI, these approaches may offer innovative strategies to mitigate long-term deleterious effects. **Dasatinib,** an FDA-approved tyrosine kinase inhibitor, and **quercetin**, a natural flavonoid, induce apoptosis in senescent cells. Their combination removes senescent cells *in vivo*, improving outcomes in fibrotic pulmonary disease, age-associated bone loss, and physical function in old age (Schafer et al., 2017; Xu et al., 2018). Quercetin benefits in rodent TBI, reducing oxidative stress, enhancing mitochondrial function, and mitigating gliosis and anxious behavior (Yang et al., 2014; Li et al., 2016; Kosari-Nasab et al., 2019). **Enzogenol**, a quercetin-containing extract, demonstrated safety, tolerance, and potential cognitive benefits for up to a year post-mild TBI (Theadom et al., 2013; Walter et al., 2017). **Curcumin**, a turmeric compound, exhibits potent senolytic activity with anti-aging effects (Beltzig et al., 2021). Curcumin analogs, **o-vanillin**, and **EF24** also display senolytic properties (Li et al., 2019). Curcumin administration reduces inflammation, glial activation, and improves cognitive outcomes in rodent TBI models (Sun et al., 2020; Khayatan et al., 2022). Additionally, curcumin protects against neurodegeneration in animal models (Ojha et al., 2012; Pluta et al., 2022) and shows benefits for critically ill TBI patients (Zahedi et al., 2021). **Luteolin**, another potential senolytic, improves cognitive function in moderate TBI patients (Campolo et al., 2021). **Fisetin**, a natural flavonol in foods like strawberries and apples, exhibits senolytic activity by targeting anti-apoptotic Bcl-2 family members (Zhu et al., 2017). Although not explored in clinical TBI, fisetin suppresses neuronal loss after experimental TBI and reduces neurodegeneration in experimental models (Zhang et al., 2018; Hassan et al., 2022). Experimental Bcl-2/Bcl-xL inhibitor **Navitoclax** (ABT263) improved cognitive function in male mice after rmTBI, indicating potential sex differences in neurodegeneration mechanisms (Schwab et al., 2022). The impact of Bcl-2 family inhibitors after moderate-severe TBI or in clinical populations remains unexplored. While promising, further research and clinical trials are necessary to establish senolytics’ mechanisms and efficacy in enhancing cognitive outcomes after TBI.

**Microglial depletion and repopulation methods** offer a promising technique for elucidating microglial biology (Barnett et al., 2021). The depletion of microglia has been investigated as an experimental therapeutic approach aimed at improving, preventing, or reversing neurocognitive deficits in various neuroinflammation-related disorders, including TBI (Dagher et al., 2015; Feng et al., 2016; Henry et al., 2020; Willis et al., 2020). The experimental removal of microglia from the CNS environment, achieved through pharmacological approaches such as administering colony-stimulating factor 1 receptor (CSF1R) inhibitors (Rice et al., 2015) or genetic induction of the diphtheria toxin receptor in microglia (Lund et al., 2018), represent a valuable tool for studying microglial contributions to TBI neuropathology. CSF1R inhibitors such as PLX5622 hinder the CSF1-dependent microglia trophic support. Consequently, administration of these inhibitors in the diet results in the depletion of over 90% of microglia *in vivo* within about 3 weeks (Rice et al., 2015). Preemptive microglial depletion, achieved through PLX5622 treatment or genetic manipulation prior to experimental TBI, has been shown to be beneficial in TBI-related spatial memory loss. This approach also stimulated functional neurogenesis in the hippocampus. However, continuous or delayed microglial depletion and repopulation failed to alleviate TBI-induced deficits, highlighting the crucial role of acutely timed or preemptive microglial treatment (Willis et al., 2020). In contrast, a separate study showed that PLX5622 administration 1 week after TBI reduced impairments in recognition and spatial memory, as well as neuropathological changes (Henry et al., 2020).

Another glia-oriented approach takes advantage of the stem cell properties of reactive astrocytes and NG2-glia, for their **direct *in vivo* reprogramming** functional neurons. Previous studies showed that some reactive astrocytes after injury may acquire stem cell-related properties, forming neurospheres *in vitro* (Lang et al., 2004; Buffo et al., 2008; Shimada et al., 2012; Guo et al., 2014). In contrast, reactive astrocytes generated glial cells (astrocytes and oligodendrocytes) but they typically did not produce neurons *in vivo* (Buffo et al., 2008; Shimada et al., 2012). Forced expression of NeuroD1 in reactive astrocytes helped overcome *trans-*lineage barriers. NeuroD1, crucial for adult neurogenesis, induces terminal neuronal differentiation (Boutin et al., 2010) and aids in reprogramming human fibroblasts into neurons (Pang et al., 2011). This theory was proven *in vitro*, when after the infection of human astrocytes by GFAP of:NeuroD1-IRES-GFP retrovirus, NeuroD1-positive cells also express positivity for NeuN, a marker of functional neurons (Guo et al., 2014). Another experiments of the same study showed that NeuroD1 may initiate a direct reprogramming of reactive glial cells post-brain injury into functional neurons in the mouse brain *in vivo*. The simultaneous generation of both excitatory and inhibitory neurons by NeuroD1 alone could potentially balance excitation and inhibition in the cortex after reprogramming (Guo et al., 2014).

Guo et al. (2014) demonstrated that the expression of NeuroD1 leads primarily to reprogramming of reactive astrocytes into glutamatergic neurons (Guo et al., 2014; Chen et al., 2020), while NeuroD1-positive NG2 cells can mature into both glutamatergic and GABAergic neurons (Guo et al., 2014). NG2 cells are major proliferative glial cells in the adult brain under normal conditions (Buffo et al., 2008; Kang et al., 2010). In the stab injury model, Guo et al. (2014) observed that the majority of cells with incorporation of NeuroD1 via CAG-GFP retrovirus were GFAP^+^, while NG2 cells created approximately 20% of the total infected cells (Guo et al., 2014). The authors suggested that different glial cells may be associated with different neuronal fate in lineage differentiation terms.

Other transcription factors, or their combinations, were also shown to induce conversion of astrocytes or NG2-glia into neurons both *in vitro* and *in vivo*: for example Neurog2 (Neurogenin2) (Heinrich et al., 2010; Grande et al., 2013), SOX2 (SRY-box transcription factor 2) (Niu et al., 2013; Heinrich et al., 2014), Ascl1 (Achaete-scute family BHLH transcription factor 1) (Liu et al., 2015; Torper et al., 2015), Brn2 (POU domain transcription factor Brn-2) (Torper et al., 2015), Myt1l (Myelin transcription factor 1 like) (Torper et al., 2015), or Dlx2 (Distal-less homeobox 2) (Heinrich et al., 2010). In contrast to the Guo et al. (2014), other studies showed that the lineage of glial cell is not crucial for determination of neuronal fate, and that different transcription factors may induce reprogramming of astrocytes or NG2-glia in both glutamatergic and GABAergic neurons (Heinrich et al., 2014; Torper et al., 2015).

The additional therapeutic targets are the SUR1 and TRPM4 channels, that play a crucial role in secondary injury processes post-TBI (see chapter Edema). The SUR1-TRPM4 complex, normally absent in the brain, becomes upregulated in various CNS cell types post-TBI (neurons, microglia, astrocytes, endothelial cells) (Simard et al., 2009; Gerzanich et al., 2019). **Targeted inhibition of the SUR1-TRPM4 complex** has demonstrated an efficacy in reducing edema and hemorrhage progression in preclinical contusional TBI models (Jha et al., 2020). Glibenclamide, also known as glyburide, acts as an antagonist for SUR1-TRPM4 and is already widely used in treating type 2 diabetes (Jha et al., 2021). Glibenclamide inhibits calcium influx, preventing depolarization and swelling in neurons, glial cells, and vascular endothelial cells following brain injury (King et al., 2018; Jha et al., 2020). Glibenclamide has proven positive effects during BBB breakdown or edema following cerebral hemorrhage, and reduces mortality in TBI, however, its impact on microglial activation and inflammatory cell infiltration remains unclear (Simard et al., 2009; Ortega et al., 2013; DeWitt et al., 2018; Shiokawa et al., 2022). While the initial research focused on the glibenclamide effect in ischemic stroke, mounting evidence underscores the central role of SUR1-TRPM4 also in secondary TBI injuries, particularly in contusional edema and hemorrhage progression (Jha et al., 2020, 2021). Glibenclamide, noted for its pleiotropic protective effects in TBI animal models, is currently the subject of a phase II clinical trial (NCT01454154). Further details can be found at https://clinicaltrials.gov/ct2/show/NCT01454154 (Lerouet et al., 2021).

Particularly noteworthy for the modern treatment of TBI can be the use of **cell therapy**, employing mesenchymal stromal cells (MSCs) or amnion-derived multipotent progenitor cells (AMPs) for enhancement of neurological recovery. The effect of administration of **AMPs** has been evaluated in PBBI model (Chen et al., 2009). The treatment significantly attenuated axonal degeneration in both the thalamus and the corpus callosum even though no significant difference in an injury volume was observed across all treatment groups. None of the labeled AMP cells appeared to express neural differentiation, as evidenced by the lack of double labeling with nestin, S-100, GFAP, and microtubule-associated protein 2 (MAP-2) immunostaining. AMP cell migration was specifically induced by PBBI and required subventricular zone homing, yet the neuroprotective effect of intracerebral ventrical treatment using AMP cells was not limited to the area where the cells were present (Chen et al., 2009). The ability of **MSCs** to promote neurite outgrowth and intrinsic neurogenesis after TBI was demonstrated in both animal models and early phase clinical studies (Zhang et al., 2015). Originally, it was thought that the recovery from brain injury following MSC therapy resulted from the replacement of damaged tissue by differentiated MSCs. However, subsequent studies offer evidence that the therapeutic effectiveness of MSCs in treating TBI is predominantly linked to the vigorous generation and release of EVs, notably exosomes (Zhang et al., 2015, 2017a,2017b; Xiong et al., 2017). Interaction of **exosomes containing miRNA** with brain parenchymal cells and the neurogenic niche promotes neurogenesis and brain remodeling, suggesting a potential benefit in the treatment of TBI. Exosomes also emerge as potent candidate biomarkers for mTBI (Atif and Hicks, 2019; Pinchi et al., 2020). Additionally, miRNAs have been identified as therapeutic targets for neurorehabilitation with reported efficacy in reducing brain edema and inflammation (Sun et al., 2018).

Overall, the study of TBI and its understanding are moving forward, with new models and techniques being developed. The future seek of new therapeutical targets should involve research of factors and signaling pathways affecting mechanisms of neuroinflammation, in both acute and chronic phases, as well as research focusing on the RNA field, specifically miRNA, which shows substantial results in other areas, including cancer studies. TBI treatment could be personalized in the future, considering the patient’s overall health, age, or sex. Additionally, a more accurate estimation of TBI, as well as a unified and precise classification (e.g., glial cell phenotype or TBI severity assessment) combined with gene analysis of individual subpopulations found in individual types of TBI, would overall contribute to the simplification of data processing and research progress acceleration.

## 14 Conclusion

Traumatic brain injury is one of the most common brain injuries with a high occurrence of mortality every year, and those who survive TBI suffer from long-term unfavorable consequences. The relevance of glial cells in TBI research has been well-known for a long time and is established by numerous published articles. This systematic review provides a comprehensive overview of the existing literature on reactive gliosis in TBI. Through a thorough analysis of numerous studies, we have synthesized the current knowledge regarding the complex cellular and molecular processes underlying reactive gliosis following TBI. Our findings highlight the multifaceted nature of reactive gliosis, showcasing its dual roles as both a neuroprotective and potentially detrimental response to TBI. While reactive gliosis may contribute to tissue repair, immune modulation, and the restoration of homeostasis, it can also lead to secondary neuroinflammation and exacerbate neurological deficits.

A deeper understanding of reactive gliosis is essential for the development of targeted therapies that can mitigate its negative effects while harnessing its neuroprotective potential. By continuing to unravel the complexities of reactive gliosis in TBI, we can strive to improve the prognosis and quality of life for individuals affected by this devastating condition. This review summarizes the key points and highlights the importance of further research in this area.

## Author contributions

ZA: Conceptualization, Funding acquisition, Visualization, Writing – original draft. MC: Conceptualization, Visualization, Writing – original draft. MA: Funding acquisition, Resources, Validation, Writing – review and editing. LV: Conceptualization, Supervision, Validation, Visualization, Writing – review and editing.

## Footnotes

1
https://www.who.int/news-room/fact-sheets/detail/dementia

2
https://health.mil/Military-Health-Topics/Centers-of-Excellence/Traumatic-Brain-Injury-Center-of-Excellence/DOD-TBI-Worldwide-Numbers

3
https://www.accessdata.fda.gov/scripts/cdrh/cfdocs/cfpmn/pmn.cfm?ID=K223602
