# Supplementary material for: Reactive gliosis in traumatic brain injury: a comprehensive review
Source: Front Cell Neurosci. 2024 Feb 28;18:1335849. doi: 10.3389/fncel.2024.1335849 (PMC10933082; doi:10.3389/fncel.2024.1335849)
Supplement: Supplementary file 1 [file Table_1.pdf]

Abbreviations:

α-syn	α-synuclein
AA	Arachidonic acid
AD	Alzheimer´s disease
ADEs	Astrocytes-derived exosomes
AHT	Abusive head trauma
AIF	Apoptosis-inducing factor
AKT	Protein kinase B
Ala	Alanine
ALS	Amyotrophic lateral sclerosis
AMPA receptor	α-Amino-3-hydroxy-5-methyl-4-isoxazole propionic acid receptor
AMPs	Amnion-derived multipotent progenitor cells
AP-1	Activator protein 1
APP	Amyloid precursor protein
AQP4	Aquaporin-4
ARE	Antioxidant response element, a cis-acting enhancer element
Arg	Arginine
ASC	Adapter protein apoptosis-associated speck-like protein containing a CARD
Ascl1	Achaete-scute family BHLH transcription factor 1
BBB	Blood-brain barrier
BDNF	Brain-derived neurotrophic factor
bFGF	Basic fibroblast growth factor
BI	Blast injury
BMP	Bone morphogenetic protein
Brn2	POU domain transcription factor Brn-2
bTBI	Blast-induced traumatic brain injury
BV2	Microglial cell line derived from C57BL/6 murine
C9ORF72	Chromosome 9 open reading frame 72
CAT	Catalase
CCI	Controlled cortical impact
CCL	CC chemokine ligand
CD68/40	Cluster of differentiation 68/40
CHI	Close-head injury
CHIMERA	Closed-Head Impact Model of Engineered Rotational Acceleration
CIG	Cornel iridoid glycoside
CNS	Central nervous system
CO	Carbon monoxide
COX-2	Cyclooxygenase 2
CREB	cAMP response element-binding protein
CSF1R	Colony-stimulating factor 1 receptor
CSPGs	Chondroitin sulfate proteoglycans
CTE	Chronic traumatic encephalopathy
CX3CL1	C-X3-C motif chemokine ligand 1
CX3CR1	C-X3-C motif chemokine receptor 1
CXCL10	C-X-C motif chemokine ligand 10

D+Q	Dasatinib and Quercetin
DAMPs	Damage-associated molecular patterns
DEGs	Differentially expressed genes
Dlx2	Distal-less homeobox 2
dpi	Days post-injury
DSCL	Deep subcortical lesion
EAAT1/2	Excitatory amino acid transporter 1/2
ECM	Extracellular matrix
EGFR	Epidermal growth factor
ERK	Extracellular signal-regulated kinase
ETC	Electron transfer chain
EV	Extracellular vesicle
FPI	Fluid percussion injury
FS	Functional seizures
FUS	Fused in sarcoma
GCS	Glasgow coma score
GFAP	Glial fibrillary acidic protein
$GFAP^+$	GFAP-positive
GLAST	Glutamate aspartate transporter
GLT-1	Glutamate transporter 1
GMF	Glia maturation factor
GMFB	Glia maturation factor β
GMFG	Glia maturation factor γ
GPx	Glutathione peroxidase
GR	Glutathione reductase
GS	Glutamine synthetase
GST	Glutathione S-transferase
H_2O_2	Hydrogen peroxide
HDAC	Histone deacetylase inhibitors
HGF	Hepatocyte growth factor
HMGB1	High-mobility group box protein 1
hMSC-EV	Human mesenchymal stem cells-derived extracellular vesicle
HO-1	Heme oxygenase-1
IBA1	Ionized calcium-binding adaptor molecule 1
IGF-1	Insulin-like growth factor-1
IKK	Inhibitory-κB kinase
IL-1α, IL-6, IL-33…	Interleukins
INF-γ	Interferon γ
iNOS	Inducible nitric oxide synthase
JAK/STAT	Janus kinase/signal transducers and activators of transcription
JNK	c-Jun N-terminal kinase
L-NAME	N(G)-nitro-L-arginine methyl ester
LPS	Lipopolysaccharide
MAP-2	Microtubule-associated protein 2
MAPK	Mitogen-activated protein kinase
MDEs	Microglia-derived exosomes

MDK	Midkine
Met/HGFR	Hepatocyte growth factor receptor encoded by the MET gene
MHCII	Major histocompatibility complex class II
MIF	Migration inhibitory factor
MMPs/MMP-9	Matrix metalloproteinases/Matrix metalloproteinase 9
MP	Microparticle
MSCs	Mesenchymal stromal cells
Mt-Rnr2	Mitochondrial gene encoding humanin protein
mTBI	Mild traumatic brain injury
MWM	Morris water maze
MyD88	Myeloid differentiation primary response 88
Myt1l	Myelin transcription factor factor 1 like
NAA	N-Acetylaspartic acid
NDEs	Neurons-derived exosomes
NF-κB	Nuclear factor κB
NFTs	Neurofibrillary tangles
NG2	Neural/glial antigen 2
NGF	Nerve growth factor
NHE1	Sodium-hydrogen antiporter 1
NKCC1	Na^+, K^+, Cl^- cotransporter
NLRP3	NLR family pyrin domain containing 3
NMDA receptor	N-methyl-D-aspartate receptor
Nrf2	Nuclear factor erythroid-derived 2 related factor 2
NO	Nitric oxide
NOR	Novel object recognition
Nox	NADPH oxidase
O2*	Superoxide radicals
ODEs	Oligodendrocytes-derived exosomes
*OH	Hydroxyl radicals
$ONOO^-$	Peroxynitrite, radical
p16INK4a	Cyclin-dependent kinase inhibitor associated with senescence
P2ry12	Purinergic receptor P2Y, G-protein coupled 12, gene
PAMPs	Pathogen-associated molecular patterns
PBBI	Penetrating ballistic-like brain injury
PC	Phosphatidylcholine
PD	Parkinson's disease
PE	Phosphoethanolamine
pMCAo	Permanent middle cerebral artery occlusion
PNES	Psychogenic nonepileptic seizures
PPAR-γ	Peroxisome proliferator-activated receptor γ
PSAP	Prosaposin
PTE	Post-traumatic epilepsy
PTN	Pleiotrophin
PTSD	Post-traumatic stress disorder
PVL	Periventricular lesions
RAC-1	Rac family small GTPase-1

RAGE	Receptor for advanced glycation end products
RNS	Reactive nitrogen species
ROS	Reactive oxygen species
rmTBI	Repetitive mild TBI
rTBI	Repetitive TBI
S100B	S100 calcium binding protein B
SA-β-Gal	Senescence-associated β-galactosidase
SASP	Senescence-associated secretory phenotype
scRNA-seq	Single-cell RNA sequencing
SMAD2	Mothers against decapentaplegic homolog 2, also known as SMAD family member 2
sMAF	Small MAF protein; musculoaponeurotic fibrosarcoma
snRNA-seq	Single-nucleus RNA sequencing
SOD1	Cu/Zn Superoxide dismutase 1
SOD	Superoxide dismutase
SOX	SRY-box transcription factor 2
ST2	Receptor suppression of tumorigenicity 2, also known as IL1RL1 or IL33R; Interleukin-1 receptor-like 1, or Interleukin-33 receptor
SUR1	Sulfonylurea receptor 1
TBI	Traumatic brain injury
TDP-43	Transactive response [TAR] DNA binding protein 43
TGF-β	Transforming growth factor β
TGFBR2	Transforming growth factor β receptor 2
TLR4	Toll-like receptor 4
TNF-α	Tumor necrosis factor α
Trf	Transferrin receptor, gene
TRPM4	Transient receptor potential melastin 4
TRPV4	Transient Receptor Potential Cation Channel Subfamily V Member 4
Tyk2	Tyrosin kinase 2
UCH-L1	Ubiquitin carboxy-terminal hydrolase L1
VEGF	Vascular endothelial growth factor
Vim	Vimentin
WD	Weight drop
YLDs	Years lived with disability
