# Supplementary material for: Reactive gliosis in traumatic brain injury: a comprehensive review
Source: Front Cell Neurosci. 2024 Feb 28;18:1335849. doi: 10.3389/fncel.2024.1335849 (PMC10933082; doi:10.3389/fncel.2024.1335849)
Supplement: Supplementary file 2 [file Table_2.pdf]

Supplementary Table 2: A list detailing various miRNAs, categorized by their functions in traumatic brain injury (TBI) and sorted according to the specific cell types from which these miRNAs originate.

| Cell Type | miRNA | Function |
|---|---|---|
| Astrocyte ADEs | miRNA-92b-3p<br>miRNA-34c<br>miRNA-361 | Protect neuron activity and alleviate nerve damage [a-d] |
| | miRNA-190b | Prevent neuronal autophagy and apoptosis [e] |
| | miRNA-7 | Downregulate the expression of NLGN2 and cause synaptic alteration [f] |
| | miRNA-873a-5p | Inhibit neuroinflammation, promote polarization of microglia, attenuate neurological deficits after TBI [g] |
| | miRNA-133b | Mediate neuron outgrowth, elongation in OGD neurons [h,i] |
| | miRNA-26a-5b | Decrease the dendritic complexity important for synaptic input [j] |
| Microglia MDEs | miRNA-124-3p | Inhibit neuroinflammation, promote neurite outgrowth, suppressing the activity of mTOR, attenuate neurodegeneration [k,l] |
| | miRNA-124<br>miRNA-137 | Exert a neuroprotective effect [m,n] |
| | miRNA-5121 | Suppress the neurite outgrowth and synapse recovery [o] |
| | miRNA-145-5p | Promote astrocyte proliferation (when downregulated) [p] |
| | miRNA-383-3p | Promote cell necroptosis (when downregulated) [r] |
| | miRNA-424-5p | Induce brain microvascular endothelial cell injury (when upregulated) [q] |


a Pei, X., Li, Y., Zhu, L., Zhou, Z., 2019. Astrocyte-derived exosomes suppress autophagy and ameliorate neuronal damage in experimental ischemic stroke. Exp. Cell Res. 382 (2), 111474 https://doi.org/10.1016/j.yexcr.2019.06.019.

b Bu, X., Li, D., Wang, F., Sun, Q., Zhang, Z., 2020. Protective role of astrocyte-derived exosomal microRNA-361 in cerebral ischemic-reperfusion injury by regulating the AMPK/mTOR signaling pathway and targeting CTSB. Neuropsychiatr. Dis. Treat. 16, 1863–1877. https://doi.org/10.2147/NDT. S260748.

c Wu, W., Liu, J., Yang, C., Xu, Z., Huang, J., Lin, J., 2020. Astrocyte-derived exosome-transported microRNA-34c is neuroprotective against cerebral ischemia/reperfusion injury via TLR7 and the NF-kappaB/MAPK pathways. Brain Res. Bull. 163, 84–94. https://doi.org/10.1016/j.brainresbull.2020.07. 013.

d Xu, L., Cao, H., Xie, Y., Zhang, Y., Du, M., Xu, X., et al., 2019. Exosome-shuttled miR-92b- 3p from ischemic preconditioned astrocytes protects neurons against oxygen and glucose deprivation. Brain Res. 1717, 66–73. https://doi.org/10.1016/j. brainres.2019.04.009.

e Pei, X., Li, Y., Zhu, L., Zhou, Z., 2020. Astrocyte-derived exosomes transfer miR-190b to inhibit oxygen and glucose deprivation-induced autophagy and neuronal apoptosis. Cell Cycle 19 (8), 906–917. https://doi.org/10.1080/15384101.2020.1731649.

f Hu, G., Niu, F., Liao, K., Periyasamy, P., Sil, S., Liu, J., et al., 2020. HIV-1 Tat-induced astrocytic extracellular vesicle miR-7 impairs synaptic architecture. J. NeuroImmune Pharmacol. 15 (3), 538–553. https://doi.org/10.1007/s11481-019-09869-8.

g Long, X., Yao, X., Jiang, Q., Yang, Y., He, X., Tian, W., et al., 2020. Astrocyte-derived exosomes enriched with miR-873a-5p inhibit neuroinflammation via microglia phenotype modulation after traumatic brain injury. J. Neuroinflammation 17 (1), 89. https://doi.org/10.1186/s12974-020-01761-0.

h Xin, H., Li, Y., Cui, Y., Yang, J.J., Zhang, Z.G., Chopp, M., 2013a. Systemic administration of exosomes released from mesenchymal stromal cells promote functional recovery and neurovascular plasticity after stroke in rats. J. Cereb. Blood Flow Metab. 33 (11), 1711–1715. https://doi.org/10.1038/jcbfm.2013. 152.

i Xin, H., Li, Y., Liu, Z., Wang, X., Shang, X., Cui, Y., et al., 2013b. MiR-133b promotes neural plasticity and functional recovery after treatment of stroke with multipotent mesenchymal stromal cells in rats via transfer of exosome-enriched extracellular particles. Stem Cells 31 (12), 2737–2746. https://doi.org/10.1002/stem.1409.

j Luarte, A., Henzi, R., Fernandez, A., Gaete, D., Cisternas, P., Pizarro, M., et al., 2020. Astrocyte-derived small extracellular vesicles regulate dendritic complexity through miR-26a-5p activity. Cells 9 (4). https://doi.org/10.3390/cells9040930.

k Huang, S., Ge, X., Yu, J., Han, Z., Yin, Z., Li, Y., et al., 2018. Increased miR-124-3p in microglial exosomes following traumatic brain injury inhibits neuronal inflammation and contributes to neurite outgrowth via their transfer into neurons. FASEB J. 32 (1), 512–528. https://doi.org/10.1096/fj.201700673R.

l Ge, X., Guo, M., Hu, T., Li, W., Huang, S., Yin, Z., et al., 2020. Increased microglial exosomal miR-124-3p alleviates neurodegeneration and improves cognitive outcome after rmTBI. Mol. Ther. 28 (2), 503–522. https://doi.org/10.1016/j. ymthe.2019.11.017.

m Song, Y., Li, Z., He, T., Qu, M., Jiang, L., Li, W., et al., 2019. M2 microglia-derived exosomes protect the mouse brain from ischemia-reperfusion injury via exosomal miR-124. Theranostics 9 (10), 2910–2923. https://doi.org/10.7150/thno.30879.

n Zhang, D., Cai, G., Liu, K., Zhuang, Z., Jia, K., Pei, S., et al., 2021. Microglia exosomal miRNA-137 attenuates ischemic brain injury through targeting Notch1. Aging (Albany NY) 13 (3), 4079–4095. https://doi.org/10.18632/aging.202373.
o Zhao, C., Deng, Y., He, Y., Huang, X., Wang, C., Li, W., 2021. Decreased level of exosomal miR-5121 released from microglia suppresses neurite outgrowth and synapse recovery of neurons following traumatic brain injury. Neurotherapeutics 18 (2), 1273–1294. https://doi.org/10.1007/s13311-020-00999-z.
p Ye, Y., Hao, J., Hong, Z., Wu, T., Ge, X., Qian, B., et al., 2021. Downregulation of MicroRNA-145-5p in activated microglial exosomes promotes astrocyte proliferation by removal of Smad3 inhibition. Neurochem. Res. https://doi.org/10.1007/s11064- 021-03446-3.
r Wei, M., Li, C., Yan, Z., Hu, Z., Dong, L., Zhang, J., et al., 2021. Activated microglia exosomes mediated miR-383-3p promotes neuronal necroptosis through inhibiting ATF4 expression in intracerebral hemorrhage. Neurochem. Res. 46 (6), 1337–1349. https://doi.org/10.1007/s11064-021-03268-3.
q Xie, L., Zhao, H., Wang, Y., Chen, Z., 2020. Exosomal shuttled miR-424-5p from ischemic preconditioned microglia mediates cerebral endothelial cell injury through negatively regulation of FGF2/STAT3 pathway. Exp. Neurol. 333, 113411 https:// doi.org/10.1016/j.expneurol.2020.113411.
